# *Plasmodium* sporozoites induce regulatory macrophages

**DOI:** 10.1371/journal.ppat.1008799

**Published:** 2020-09-08

**Authors:** Béatrice M. F. Winkel, Leonard R. Pelgrom, Roos van Schuijlenburg, Els Baalbergen, Munisha S. Ganesh, Heleen Gerritsma, Clarize M. de Korne, Nikolas Duszenko, Marijke C. C. Langenberg, Séverine C. Chevalley-Maurel, Hermelijn H. Smits, Esther C. de Jong, Bart Everts, Blandine Franke-Fayard, Meta Roestenberg

**Affiliations:** 1 Department of Parasitology, Leiden University Medical Center, Leiden, The Netherlands; 2 Interventional Molecular Imaging Laboratory, department of Radiology, Leiden University Medical Center, Leiden, The Netherlands; 3 Department of Experimental Immunology, Amsterdam UMC, location AMC, Amsterdam, The Netherlands; 4 Department of Infectious Diseases, Leiden University Medical Center, Leiden, The Netherlands; Burnet Institute, AUSTRALIA

## Abstract

Professional antigen-presenting cells (APCs), like macrophages (Mϕs) and dendritic cells (DCs), are central players in the induction of natural and vaccine-induced immunity to malaria, yet very little is known about the interaction of SPZ with human APCs. Intradermal delivery of whole-sporozoite vaccines reduces their effectivity, possibly due to dermal immunoregulatory effects. Therefore, understanding these interactions could prove pivotal to malaria vaccination. We investigated human APC responses to recombinant circumsporozoite protein (recCSP), SPZ and anti-CSP opsonized SPZ both in monocyte derived MoDCs and MoMϕs. Both MoDCs and MoMϕs readily took up recCSP but did not change phenotype or function upon doing so. SPZ are preferentially phagocytosed by MoMϕs instead of DCs and phagocytosis greatly increased after opsonization. Subsequently MoMϕs show increased surface marker expression of activation markers as well as tolerogenic markers such as Programmed Death-Ligand 1 (PD-L1). Additionally they show reduced motility, produce interleukin 10 and suppressed interferon gamma (IFNγ) production by antigen specific CD8^+^ T cells. Importantly, we investigated phenotypic responses to SPZ in primary dermal APCs isolated from human skin explants, which respond similarly to their monocyte-derived counterparts. These findings are a first step in enhancing our understanding of pre-erythrocytic natural immunity and the pitfalls of intradermal vaccination-induced immunity.

## Author summary

Malaria continues to be the deadliest parasitic disease worldwide, and an effective vaccine yielding sterile immunity does not yet exist. Attenuated parasites can induce sterile protection in both human and rodent models for malaria, but these vaccines need to be administered directly into the bloodstream in order to convey protection; administration via the skin results in a much-reduced efficacy. We hypothesized this is caused by an early immune regulation initiated at the first site of contact with the immune system: the skin. However, the human skin stage of malaria has not been investigated to date. We used human antigen presenting cells as well as whole human skin explants to investigate (dermal) immune responses and found that *Plasmodium* sporozoites are able to suppress immune responses by inducing regulatory macrophages. Our study provides new insights in the mechanism of early immune regulation exploited by *Plasmodium* parasites and can help to explain why intradermal vaccination using whole attenuated sporozoites results in reduced protection.

## Introduction

Despite decades of research into the world’s most deadly parasite, over 200 million individuals develop malaria after the bite of an infected *Anopheles* mosquito yearly[1]. Unfortunately, natural immunity to malaria mounts only very slowly and requires sustained exposure to repeated infections[2]. The only currently licensed malaria vaccine, RTS,S, is based on circumsporozoite protein (CSP), the most abundant and immunodominant protein on the surface of malaria sporozoites (SPZ), and yields only limited protection[3, 4]. Therefore, the development of highly effective vaccines remains of vital importance. Attenuated malaria sporozoites that arrest development in the liver are promising vaccine candidates because they yield sterile protection against homologous parasite challenge in malaria naïve individuals[5, 6]. The protection of these attenuated SPZ vaccines is thought to be mediated through interferon gamma (IFNγ)-producing CD8^+^ effector T cells which target infected hepatocytes[7, 8], as well as antibodies which target the migrating extracellular sporozoite on its way to the liver[9–11]. Large numbers of sporozoites seem to be required to induce sufficient protective immune responses in attenuated SPZ vaccination, whereby there is a remarkable difference between intradermal (ID), intravenous (IV) or mosquito bite administration[5, 12–14]. Given the inferiority of ID administration, the immune interaction between attenuated SPZ and skin immune cells might downmodulate the ensuing immune responses. Evidence for this hypothesis was found in mice, where lower levels of protection after ID vaccination were associated with an increase in immune regulatory interleukin (IL)-10 producing lymphocytes in the skin draining lymph node (sdLN)[12]. Understanding the interplay between SPZ and the immune system is important to overcome malaria tolerogenic responses and potentially enhance the potency of attenuated SPZ to allow for skin-administered vaccines.

It was previously thought that SPZ rapidly leave the skin, outrunning host immune cells due to their high migratory velocity[15]. However, recent research has shown that only a minority of injected SPZ succeed in finding a blood vessel to transport them to the liver, and that the majority of injected parasites remains in the skin[16–18]. There, SPZ interact with antigen-presenting cells (APCs) such as dermally-residing dendritic cells (DCs) and macrophages (Mϕs)[19], which orchestrate immune responses by providing processed antigens, co-stimulatory molecules and cytokines to T lymphocytes[20–24]. Ultimately, SPZ were shown able to affect skin regulatory T cell (Treg) activity in mice[25]. How malaria SPZ initiate these responses has not been investigated to date. We hypothesize that SPZ enhance their chance of survival by inducing a regulatory immune environment through exploitation of the signaling mechanisms of APCs.

Plausible candidate targets for exploitation of APC pathways are immune checkpoints. Checkpoint molecules such as Programmed Death-1 (PD-1) and its ligand (PD-1/PD-L1) are regulators of immune activation and have initially been described in the context of tumor immunology[26–29]. PD-L1 is expressed on APCs and, after ligation with PD-1 on T-cells, can lead to T-cell anergy[26, 30, 31] and the induction of immunological tolerance[27–29]. Many pathogens such as viruses[32], bacteria[33] as well as parasites[34–38] have been demonstrated to use immune checkpoints to their advantage. Blood stage malaria parasites were shown to reduce T cell immunity through induction of the PD-L1 pathway[39]. Whether SPZ are able to similarly skew dermal APC responses to induce tolerance at the pre-erythrocytic stage of disease is still unknown.

**Fig 1:**
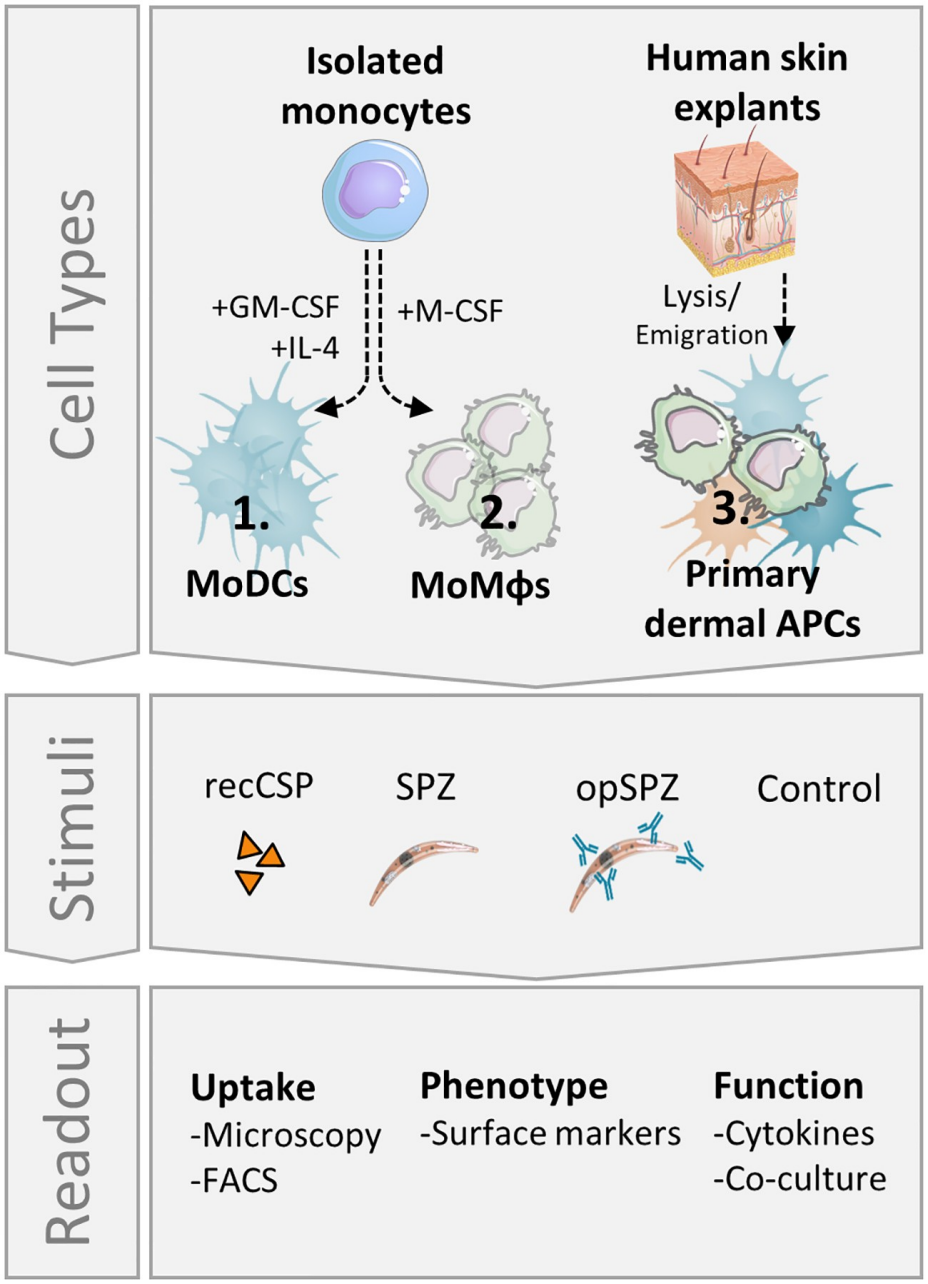
Experimental setup. MoDCs and MoMϕs were differentiated *in vitro* from freshly isolated monocytes. Additionally, fresh human skin explants containing primary dermal APCs were lysed to form a single cell suspension. All cell types were stimulated *in vitro* with recombinant SPZ surface antigen, circumsporozoite protein (recCSP) or whole sporozoites (untreated: SPZ or opsonized: opSPZ). Uptake of recCSP and (op)SPZ was determined by confocal microscopy and flow cytometry. Subsequently, cells were analyzed for their phenotype and function. Images of skin and sporozoite were reproduced. They were purchased for publication at Turbosquid.com.

To date, studies investigating the malaria skin stage have focused on rodent models. However, using rodent parasites in rodent skin in lieu of human parasites in human skin poses obstacles for extrapolating the findings to humans. Mouse skin is both anatomically[40] as well as immunologically[21, 41, 42] different from human skin. In addition, some rodent malaria parasites are able to invade and develop inside skin cells[43], whereas for human parasites this is still unclear. Therefore, we set out to investigate the interaction of *Plasmodium falciparum* (*Pf*) SPZ with human (skin) APCs (Fig 1) and analyze their effect on the ensuing adaptive immune response.

## Materials and methods

### Parasite culture

*Plasmodium berghei* (*Pb*) SPZ were obtained from a transgenic rodent malaria species that expresses fluorescent and luminescent reporter proteins: *Pb* line 1868cl1 expressing mCherry and luciferase under the constitutive HSP70 and *eef1a* promotors respectively[44] (RMgm-1320, www.pberghei.eu) and *Pb* line Bergreen[45], expressing green fluorescent protein (GFP) under the constitutive HSP7*0* promoter (RMgm-757, www.pberghei.eu). Mosquitoes were infected with *Pb* by feeding on infected mice as described previously[46]. We used female OF1 mice (6–7 weeks old; Charles River, Leiden, The Netherlands). In addition, SPZ were obtained from the human parasite *Plasmodium falciparum* (*Pf*; NF54[47]; WT or mCherry-expressing under the Sui1 promotor). Mosquitoes were infected with *Pf* by standard membrane feeding as previously described[48]. Salivary glands of infected and uninfected mosquitoes were manually dissected at day 21–28 (*Pb*) or day 14–21 (*Pf*) post infection. Salivary glands were kept on ice until use within 1 hour. Immediately prior to their use, glands were homogenized to extract parasites. Parasites were counted using a Bürker chamber.

### Opsonization of parasites

Opsonization of parasites was performed by incubating parasite sporozoites with anti-CSP antibody (For *Pf* SPZ: 2A10; IgG2ακ; 10 μg/ml; MR4, BEI resources, for *Pb* SPZ: 3D11; 70ug/ml; kindly provided by Prof. M. Prudêncio) for 30 minutes at room temperature before stimulation.

### Monocyte-derived dendritic cells (MoDCs) and macrophages (MoMϕ) culture

Monocytes were isolated from venous whole blood of healthy volunteers using CD14^+^ MACS isolation (Miltenyi Biotec, Bergisch Gladbach, Germany) and differentiated into MoDCs as described previously[49]. Alternatively, monocytes were differentiated into M0 MoMϕ using Macrophage Colony Stimulating Factor (M-CSF; 20ng/ml; Biolegend, San Diego, CA, USA)[50]. On Day 6, MoDCs and M0 MoMϕ were harvested, counted and re-cultured at 10^5^ cells/24-well NUNC plate with Nunclon delta surface coating in RPMI containing 10% FCS supplemented with Penicillin/streptomycin and rested for 24 hours. For recCSP and SPZ uptake assays the cells were re-cultured at 10^4^ cells/96-well.

### Sporozoite and recCSP uptake analysis by flow cytometry

For uptake experiments, rested MoDCs and MoMϕs were stimulated for 1 hour with recCSP in the concentrations described or a 1:1 sporozoite:cell number of (opsonized) fluorescent *Pb* parasites. Opsonization of parasites was performed as described above. Alternatively, SPZ were fixed for 15 minutes at room temperature using 4% formaldehyde, which was followed by three washes with phosphate buffered saline (PBS). RecCSP had been fluorescently labelled using a commercial antibody labeling kit (ALEXA Fluor 660 antibody labeling kit, Thermo Fisher Scientific, Waltham, MA, USA) according to their protocol. After 1 hour of stimulation, cells were harvested, stained with 7-aminoactinomycin D (7-AAD) live/dead dye (Invitrogen, Waltham, MA, USA) and analyzed by Flow Cytometry using a BD LSR Fortessa (BD Biosciences, San Jose, CA, USA) and analyzed in FlowJo version 9.9.6 (FlowJo LLC, Ashland, OR, USA).

### Confocal microscopy uptake analysis of Pf SPZ

Four-quadrant glass bottom confocal dishes (ø14mm cover slip; MatTek Corporation) were coated overnight with Poly-D-Lysine (PDL) at 37°C. 10^4^ MoDCs and MoMacs were plated into each quadrant and allowed to adhere for 6 hours. (Opsonized) mCherry expressing *Pf* SPZ were added in a 1:1 ratio and fixed after 1 hour using 15 minute incubation with 3.7% formalin (Sigma Aldrich, St Louis, MO, USA) at room temperature. Opsonization of parasites was performed as described above. Dishes were imaged in 3-dimensional Z-stacks using the 40x objective on a SP8X WLL (white light laser) microscope (Leica Microsystems, Wetzlar, Germany; 4 donors, 12–16 fields of view per donor per condition; on average 1150 MoMacs and 550 MoDCs per donor per condition). The nuclei of the cells were stained with Hoechst prior to the imaging. mCherry was excited at 587 nm and the emission was collected between 600–650 nm. Hoechst was excited at 405 nM and emission was collected between 420–470 nM. Cell membranes were identified either on the basis of brightfield or after 10 minute incubation with Cellbrite Green (Biotium, Hayward, CA, USA). CellBrite Green was excited at 488 nm and emission collected between 500–530 nm. Cells were analyzed manually for intracellular SPZ using LASX software (Leica Microsystems).

### Confocal video microscopy of whole SPZ phagocytosis

Glass bottom confocal dishes (ø14mm cover slip; MatTek Corporation) were coated overnight with Poly-D-Lysine (PDL) at 37°C. 10^4^ MoDCs and MoMacs were plated onto dishes and allowed to adhere for 6 hours. The nuclei of the cells were stained with Hoechst prior to the imaging. Opsonized mCherry expressing *Pb* or *Pf* SPZ added in a 1:1 ratio and dishes were imaged directly using the 40x objective on a SP8X WLL (white light laser) microscope (Leica Microsystems, Wetzlar, Germany) with climate control at 37°C, 5% CO_2_ for the duration of 1 hour. mCherry was excited at 587 nm and the emission was collected between 600–650 nm. Hoechst was excited at 405 nM and emission was collected between 420–470 nM.

### Sporozoite and recCSP stimulation

On day 7, rested immature MoDCs and M0 MoMϕ were exposed for 24 hours to recombinant *pf* circumsporozoite protein (recCSP; Alpha Diagnostics, San Antonio, TX, USA catalognr: CSPF17-R-10), 20.000 *Plasmodium falciparum* (Pf) SPZ, a matched volume of salivary gland extract (SGE) of uninfected control mosquitoes or medium as a true negative control and ultrapure lipopolysaccharide (LPS; 100ng/ml; *Escherichia coli* 0111 B4 strain, InvivoGen, San Diego, CA, USA) as an inflammatory control. When different well sizes were used, the same 1:5 sporozoite:cell ratio was maintained. Importantly, SGE was used as a matched control as SPZ preparations do contain traces of salivary gland material. After 24 hour, cells were harvested, stained with 7-aminoactinomycin D (7AAD) live/dead dye (Invitrogen, Waltham, MA, USA), and antibodies against CD80 (L307.4; BD Biosciences), CD86 (N331 FUN-1; BD Biosciences), CD25 (2A3; BD Biosciences), CD197 (3D12; BD Biosciences), CD206 (15–2; Biolegend), CD209 (DCN46; BD Biosciences), PD-L1 (MIH1; BD Biosciences), PD-L2 (MIH18; eBiosience) and Immunoglobulin-like transcript 3 (ILT3; zm4.1; Biolegend) and analyzed by Flow Cytometry using a BD LSR Fortessa (BD Biosciences, San Jose, CA, USA) and analyzed in FlowJo version 9.9.6 (FlowJo LLC, Ashland, OR, USA).

### Naïve CD4+ T cell co-culture

For analysis of T cell polarization, 5x10^3^ LPS-matured, *Pf* SPZ or control antigen-stimulated MoDCs were co-cultured with 2x10^4^ allogeneic naïve CD4+ T cells isolated from buffy coat (Sanquin, Amsterdam, The Netherlands). Co-cultures were performed in the presence of staphylococcal enterotoxin B (10pg/ml)[49]. On days 6 and 8, recombinant human IL2 (10U/ml; R&D Systems) was added and the T cells were expanded until day 11. Intracellular cytokine production was analyzed after polyclonal restimulation with 100ng/ml phorbol myristate acetate (PMA; Sigma Aldrich) and 1ug/ml ionomycin (Sigma Aldrich) for 6 hours. Brefaldin A (10ug/ml; Sigma Aldrich) was added for the last 4 hours of restimulation. Cells were fixed in 3.7% paraformaldehyde (Sigma Aldrich), permeabilized with permeabilization buffer (Affymetrix, Santa Clara, CA, USA), stained with antibodies against IL-4 and IFNγ (BD biosciences) and analyzed with flow cytometry using a FACScanto (BD Biosciences) with FlowJo version 9.9.6 (FlowJo LLC). In addition, 10^5^ expanded CD4 T cells were restimulated with antibodies against CD3 and CD28 (Biolegend) for 24 hours in a 96-wells plate. Supernatants were harvested and analyzed for IL-10 secretion using standard ELISA (Sanquin, Amsterdam, The Netherlands).

### Cytokine measurement

MoDC and MoMϕ supernatants were harvested after 24 hour stimulation with *Pf* SPZ, SPZ antigens or controls. Supernatants (IL-10, IL-1β and IL6) were analyzed by standard ELISA (Sanquin, The Netherlands).

### Wound closing assay

MoMϕs were plated in a monolayer in a 96 wells flat bottom plate and allowed to adhere for 24 hours. *Pf* SPZ (1:1 ratio), matched equivalent of SGE or Cytochalasin D[51] (10uM) was added, and plates were spun down at a low centrifugal speed (1000rpm) in order to sediment SPZ. Using a pipet tip a scratch was made in one fluent motion in the monolayer. Plates were incubated at 37°C, 5% CO_2_ and imaged after 1 hour and 40 hours using a SP8 inverted confocal microscope (Leica Microsystems, Wetzlar). Images were analyzed using ImageJ 1.48v public domain software (NIH, USA) using the following protocol: Process-> Find edges, process->Sharpen, Image-> Adjust threshold (set threshold manually to reduce background noise), process->Find edges, analyze particles->plot profile. Profile data of scratch area of the image was then loaded into Microsoft Excel and plotted using GraphPad Prism (La Jolla, CA, USA) version 7. The scratch area was located a gray value below 700. In addition, we plotted the difference between the mean gray value over a representative section of the full scratch area per donor comparing T40 with T1.

### CSP specific CD8 T cell co-culture

A CSP specific CD8^+^ T cell clone[52] was expanded in co-culture with irradiated PBMC feeder cells from anonymous HLA-matched donors (3000 rad; 10:1 (feeder:CD8 cells)) and irradiated Epstein-Barr virus immortalized B cells (3000 rad; 2:1 (EBV to CD8 cells); mix of three, AKO, Boleth and JY cells) in RPMI containing 10% heat inactivated human serum, β-mercaptoethanol (50u; Sigma-Aldrich, St Louis, MO, USA)), phytohemagglutinin (1ug/ml; Thermo Fischer Scientific, Waltham, MA, USA) and recombinant human IL-2 (100U/ml; R&D systems, Minneapolis, MI, USA) in a 96 wells round bottom plate. After 10 days, cells were harvested, counted and expanded further by co-culture of 2x10^4^ CD8 cells with 5x10^4^ irradiated feeder PBMCs, human recombinant IL-15 (5ng/ml), human recombinant IL-7 (5ng/ml), CD3/28 Dynabeads (Thermo Fisher Scientific) in RPMI 10% human serum. After two days, human recombinant IL-2 was added (100U/ml), cells were used 10 days after expansion.

T cells were cultured together with peptide-stimulated MoDCs overnight in the presence or absence of *Pf* SPZ or control antigen-stimulated MoMϕs (40.000 T cells, 10.000 MoDCs and 10.000 MoMϕs) in a 96 wells round bottom. For blocking of the IL10 and PD-1 pathway, antibodies against IL-10 (Biolegend, clone JES3-19F1) and IL10 receptor (CD210, Biolegend clone 3F9) or PD-1 (pembrolizumab) were added during the incubation in a concentration of 10ug/ml. After 4 hours, Brefaldin A (10ug/ml; Sigma Aldrich) was added. Cells were harvested, stained with Aqua fixable live/dead dye (Thermo Fischer Scientific) and fixed in 3.7% paraformaldehyde (Sigma Aldrich). After permeabilization with permeabilization buffer (Affymetrix, Santa Clara, CA, USA) cells were stained intracellularly for CD3 (UCHT1; eBioscience, Santa Clara, CA, USA), IFNγ (B27; BD Biosciences), CD137 (4B4-1; Biolegend), Granzyme A (CB9; Biolegend), Granzyme B (GB11; Biolegend) and Perforin (dG9; Invitrogen) and analyzed by Flow Cytometry using a FACSCanto II (BD bioscience).

### Suppression assay

MoDCs were generated and stimulated as described above. Next we analyzed suppression of proliferation of bystander T cells by test T cells after interaction with *Pf* SPZ-stimulated MoDCs. SPZ stimulated, LPS-matured MoDCs were co-cultured with naïve CD4^+^ T cells for 6 days in a 24 wells plate in a 1:10 ratio (Test T cells). Next, mature test T cells were harvested, irradiated (30Gy) to prevent proliferation, and co-cultured with carboxyfluorescein succinimidyl ester (CFSE) stained CD4^+^ memory T cells of the same T cell donor in a 1:2 ratio (25.000 and 50.000 respectively) in the presence of 1000 LPS matured MoDCs.

Vitamin D treated MoDCs were used as a regulatory T cell-inducing control. After 6 days of co-culture, bystander (target) T cells were harvested and stained with CD45RO, CD4, CD25 and Aqua live dead stain (Invitrogen). Samples were analyzed with flow cytometry using a FACScanto II (BD Biosciences) with FlowJo version 9.9.6 (FlowJo LLC). Cell proliferation was calculated by measuring the CFSE peaks using the Proliferation Tool by FlowJo. Data was analyzed in Graphpad Prism version 7.

### Skin explants

Human skin explants were obtained from collaborating hospitals immediately after abdominal or breast skin reduction surgery (IRB B18.009) and kept at 4°C until use (within 6 hours). Six biopsies, each 6mm in diameter, were taken using punch biopsies. Biopsies were either cultured for 72 hours, floating in medium supplemented with 10% FCS after which biopsies were discarded and supernatant was spun down to collect spontaneously emigrated cells (dermal APCs; used for confocal quantification of *Pf* SPZ uptake), or enzymatically digested overnight using the A and D enzymes of the human Whole Skin Dissociation Kit with associated C-tubes and GentleMACS tissue dissociator (all Miltenyi Biotec, Bergisch Gladbach, Germany) for immunological assays.

### Confocal microscopy Pf SPZ uptake by dermal APCs

Emigrated dermal APCs were subsequently frozen and stored in liquid nitrogen. For confocal uptake experiments, thawed cells were plated in with Poly-D-Lysine coated glass bottom confocal dishes (ø14mm cover slip; MatTek Corporation) and allowed to adhere for 6 hours. Cells were stimulated with (opsonized) *Pf* SPZ (1:1 SPZ:cell ratio) or SGE control and fixed after 1 hour using 15 minute 3.7% formalin (Sigma Aldrich, St Louis, MO, USA) incubation at room temperature. Cell membranes were stained using 10 minute incubation with CellBrite Green (Biotium). Nucleic dye Hoechst was added prior to imaging. Dishes were imaged in 3 dimensional Z-stacks using the 40x objective on a SP8X WLL (white light laser) microscope (Leica Microsystems; 3 donors, 20 fields of view per donor per condition). mCherry was excited at 587 nm and the emission was collected between 600–650 nm, CellBrite Green was excited at 488 nm and emission collected between 500–530 nm and Hoechst was excited at 405 nm and emission collected between 420–470 nm. Cells were analyzed for intracellular SPZ manually using LASX software (Leica Microsystems).

### Phenotypic analysis of dermal APCs after SPZ uptake

Lysed single cell skin suspensions were counted and plated out in 48 well plates at 2*10^5^ cells per well. Cells were stimulated with mCherry expressing *Pb* or Wild-type *Pf* SPZ for 1 hour (*Pb* uptake experiments) or 24 hours (*Pb* and *Pf* stimulated dermal APC phenotyping). Cells were stained with 7AAD live/dead dye (uptake) or with Aqua fixable live/dead dye (Thermo Fischer Scientific; phenotyping), CD45 (HI30; Biolegend), HLA-DR (L243; eBioscience), CD11c (Bu15, Biolegend), PD-L1 (MIH1; BD Biosiences), CD80 (L307.4; BD Biosciences) and analyzed by Flow Cytometry using an LSRFortessa (BD Biosciences). Data was analyzed in FlowJo version 9.9.6 (FlowJo LLC). Gates were set using ‘fluorescence minus one’ (FMO) stained control samples.

### Statistical analysis

Data was analyzed using GraphPad Prism (La Jolla, CA, USA) version 7 or 8. Comparisons between two or more independent data groups were made by Student’s T test (parametric data)/ Wilcoxon test (nonparametric data) or analysis of variance test (ANOVA) respectively. P<0.05 was considered statistically significant.

### Ethics statement

All animal experiments of this study were granted with a license by Competent Authority after an advice on the ethical evaluation by the Animal Experiments Committee Leiden (AVD1160020173304-01). All experiments were performed in accordance with the Experiments on Animals Act (Wod, 2014), the applicable legislation in the Netherlands and the European guidelines (EU directive no. 2010/63/EU) regarding the protection of animals used for scientific purposes. All experiments were executed in a licensed establishment for use of experimental animals (LUMC).

The use of human skin explants (obtained as waste material after abdominal reduction surgery) for this research was approved by the Commission Medical Ethics (CME) of the LUMC, Leiden. Approval number CME: B18-009.

## Results

### Uptake of RecCSP and SPZ by APCs

**Fig 2:**
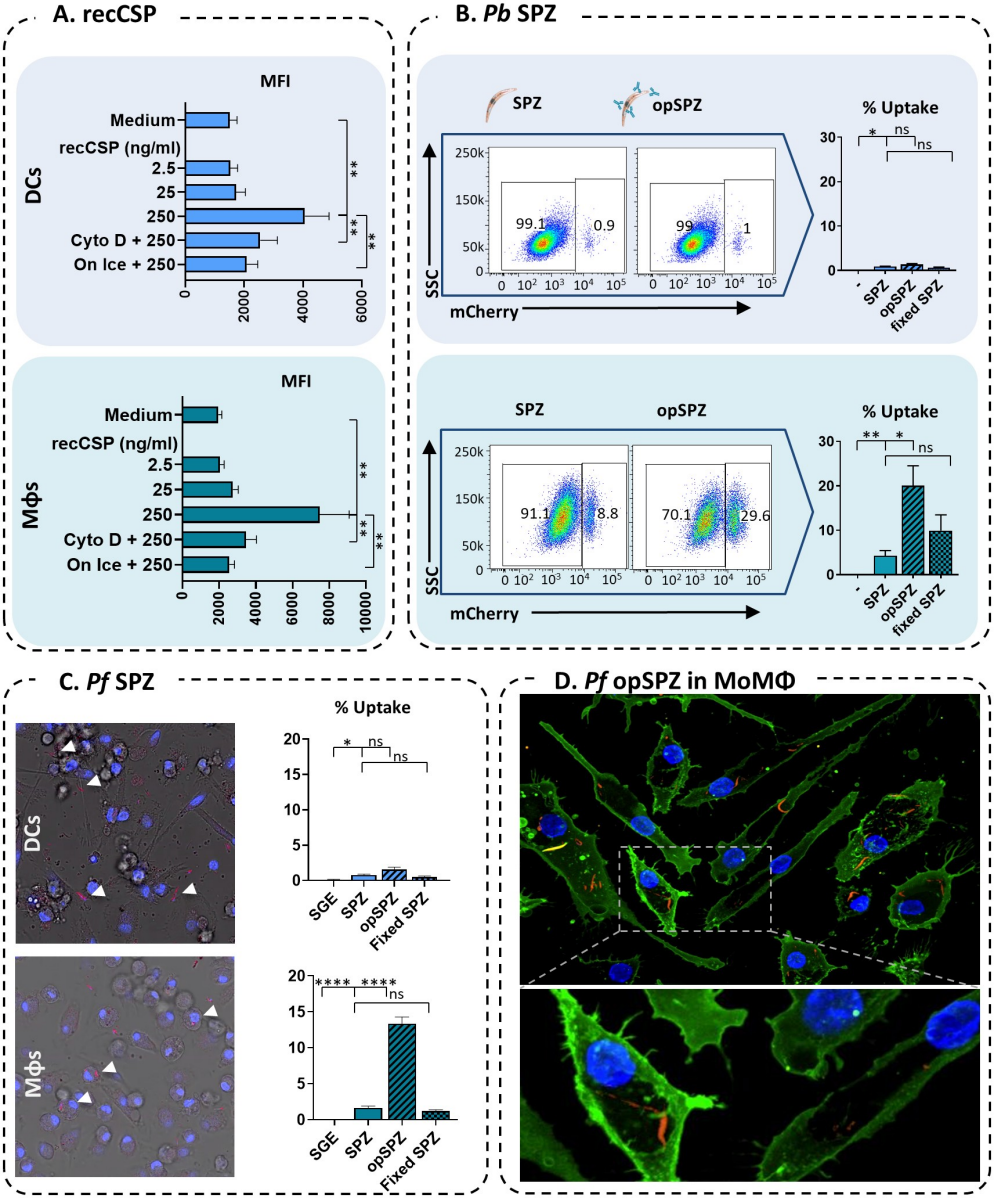
Uptake of recCSP and whole SPZ by MoDCs and MoMϕs. **A**. Quantification of fluorescent recCSP uptake (ng/ml) in MoDCs (top) and MoMϕs (bottom); MFI: median fluorescence intensity. N = 2, 4 donors. **B**. Uptake of whole *Pb* sporozoites by MoDCs and MoMϕs. Representative flow cytometry plots showing mCherry expression in MoDCs (top) and MoMϕs (bottom) after stimulation with SPZ or opsonized SPZ using an anti-CSP antibody (opSPZ). Quantification of SPZ uptake (% of mCherry^+^ APCs) by flow cytometry. N = 3, 9 donors (fixed SPZ: 4 donors). **C**. Uptake of whole *Pf* sporozoites by MoDCs and MoMϕs quantified by confocal microscopy. N = 2, 4 donors, on average 1150 MoMacs and 550 MoDCs per donor per condition. **D**. Confocal microscopy image of *Pf* SPZ uptake by MoMϕs.

We first determined which APC types take up CSP and/or whole SPZ. We found that fluorescently labeled, recombinant CSP (recCSP) was taken up readily by both monocyte-derived dendritic cells (MoDCs) as well as M0 monocyte-derived macrophages (MoMϕs) *in vitro* in a concentration dependent manner (Fig 2A). In contrast, *Plasmodium berghei* (*Pb*) SPZ were preferentially phagocytized by MoMϕs. Whereas only about one percent (mean 0.86%; range 0.62–1.36) of MoDCs phagocytized mCherry-expressing *Pb* SPZ, on average four percent (range 0.78–10.2%) of MoMϕs took up whole SPZ *in vitro* (Fig 2B, S1 Movie). Opsonization of SPZ using an anti-CSP antibody increased their uptake by MoMϕs five-fold (mean 20%; range 9.5–37.5), whereas uptake by MoDCs was unaffected. Flow cytometric analysis of *Plasmodium falciparum (Pf)* uptake was not feasible due to the low fluorescent signal of the transgenic *Pf* lines used in comparison to the high autofluorescence of human APCs (S1 Fig). Therefore, we quantified *Pf* uptake by confocal microscopy. Similarly, *Pf* SPZ are were phagocytized by MoMϕs, but not MoDCs (Fig 2C; MoMϕs: mean 3.2%, range 0–9.7%; MoDCs: mean 0.76%, range 0–5.1%; S1 and S2 Movies) and uptake by MoMϕ, but not MoDCs, increased 3.6 fold after parasite opsonization (mean 11.6%, range 2–29.5%). SPZ were clearly identified on their morphology and fluorescence and membrane staining of APCs allowed us to determine their intracellular location (Fig 2D). In addition, video microscopy revealed that uptake of SPZ by MoMϕs is an active phagocytic process and not the result of SPZ invasion of the cells (S1 Movie). Furthermore, formaldehyde fixation of non-opsonized SPZ, which renders SPZ immobile and incapable of invading, did not decrease uptake of SPZ by MoMϕs, suggesting that increased uptake of opsonized-SPZ by MoMϕs is not due to immobilization or a result of active APC invasion.

### MoDCs and MoMϕs do not respond to recCSP stimulation

**Fig 3:**
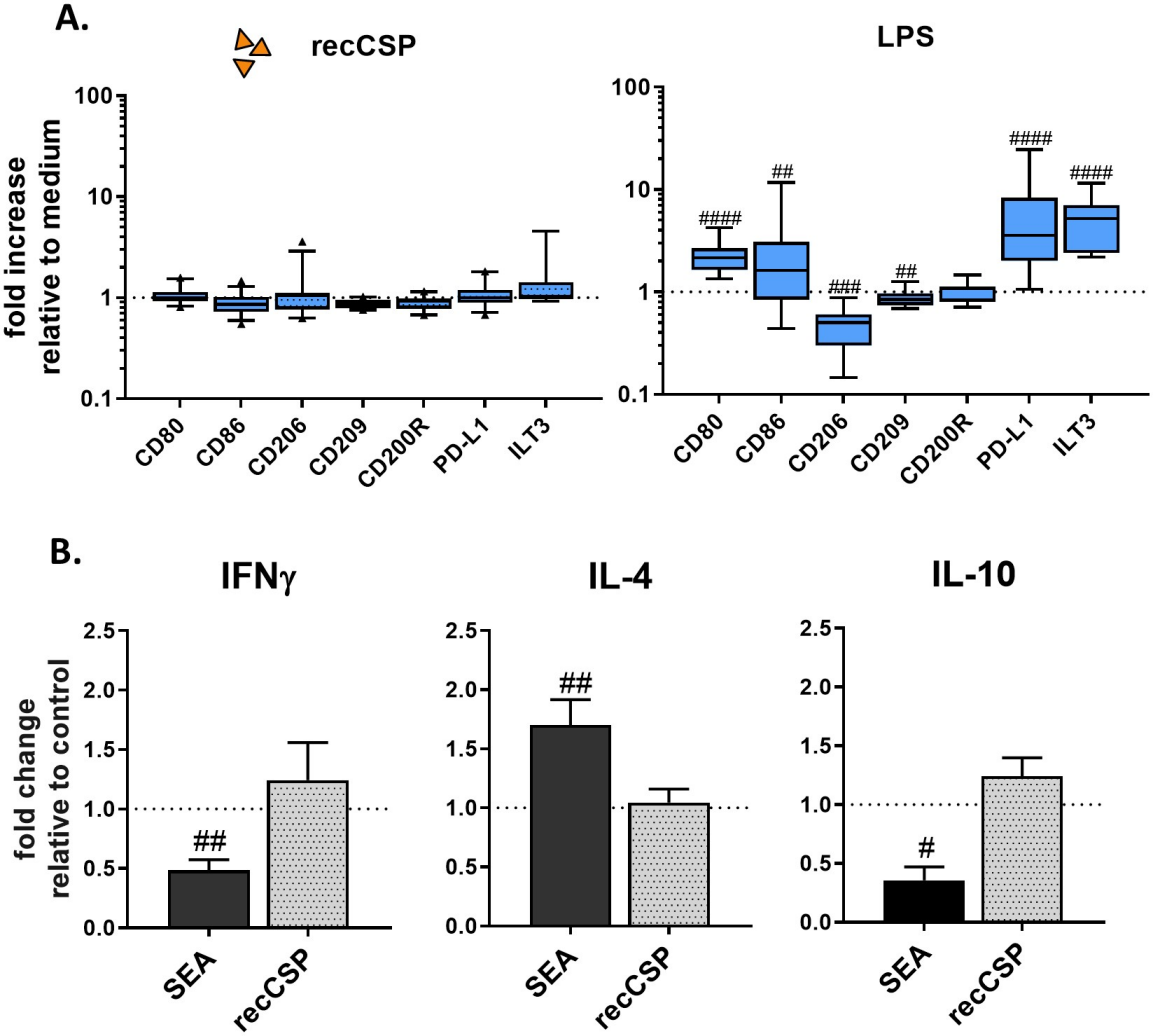
MoDC responses to recCSP. **A**. RecCSP stimulation (250 ng/ml) does not alter MoDC surface markers. Responses to control LPS are shown on the right. Data shown as fold changes compared to medium stimulated control. N = 8, 10–19 donors per marker. **B**. CD4^+^ T cell polarization after recCSP stimulation. RecCSP stimulation of LPS-matured MoDCs does not polarize naïve T cells towards a Th1 (IFNγ), Th2 (IL-4; both measured by intracellular staining, N = 2, 6 donors.) or Treg (IL-10; measured by ELISA after CD3/28 restimulation, N = 2, 4 donors) response. Soluble Schistosome Egg Antigen (SEA) used as a Th2 inducing control. Data shown relative to LPS-matured MoDC control. A and B: # indicates comparison to control. #: P = <0.05, ##: P = <0.005, ###: P = <0.0005 and ####: P = <0.0001.

**Fig 4:**
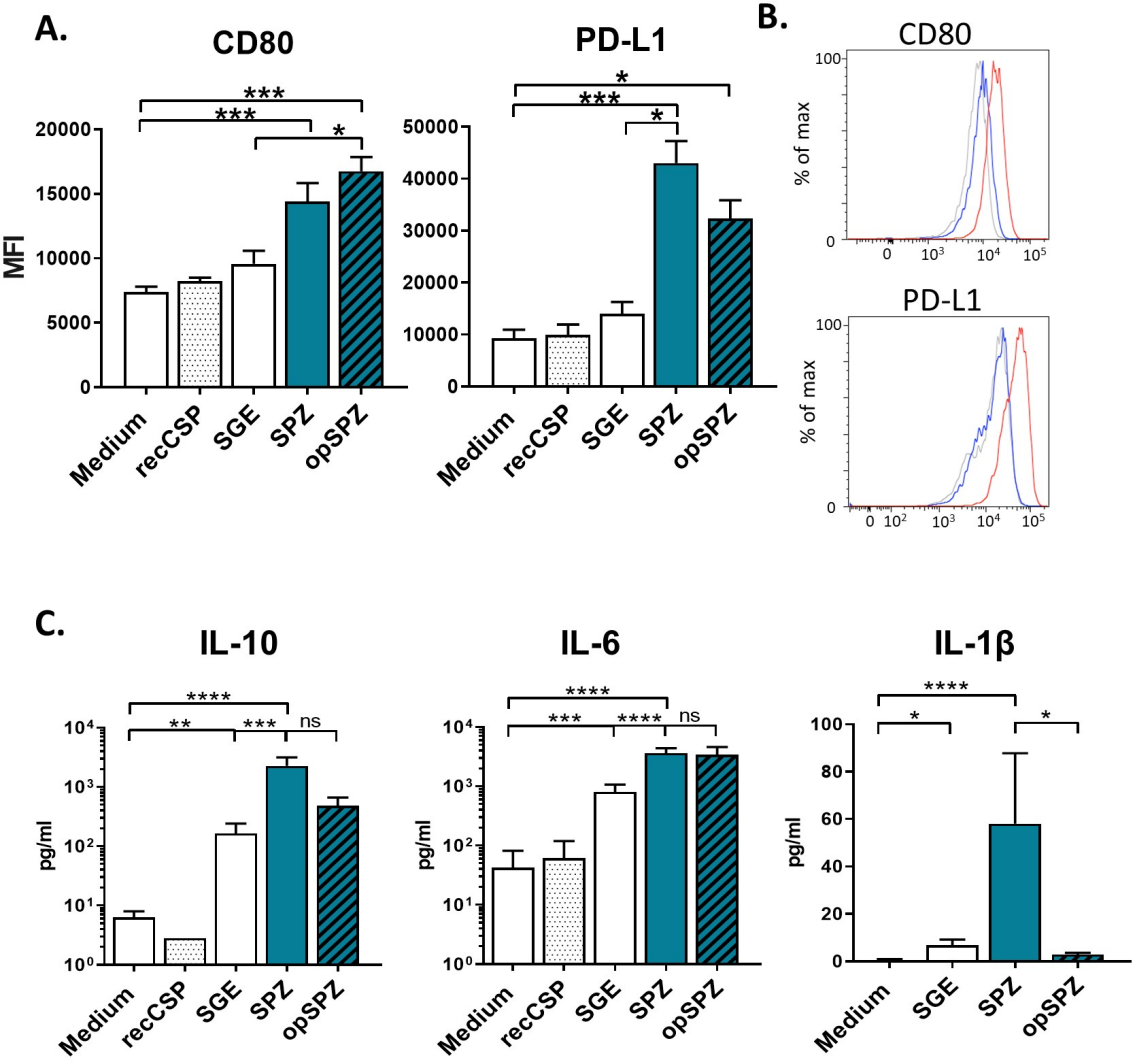
MoMϕ responses to (opsonized) *Pf* SPZ. **A**. SPZ and opSPZ upregulate activation marker CD80 as well as regulatory marker PD-L1. Analysis using one way ANOVA. N = 9, at least 20 donors. **B**. Representative histograms of CD80 and PD-L1 surface marker expression. Medium stimulated MoMϕs in grey, SGE stimulated MoMϕs in blue and SPZ stimulated MoMϕs in red. **C**. MoMϕs produce IL-10, IL-6 and IL1β in response to (op)SPZ stimulation. Analysis using one way ANOVA on log transformed data. *: P = <0.05, **: P = <0.005, ***: P = <0.0005 and ****: P = <0.0001, ns = not significant. IL-10: N = 8, 25 donors; IL-6: N = 5, 17 donors; IL1β: N = 3, 7 donors.

Having shown that MoDCs readily take up recombinant *Pf* CSP (recCSP), but almost no SPZ, we next investigated their phenotypic and functional response to the most abundant surface protein of SPZ: CSP (the main component of RTS,S vaccine, which is excreted upon SPZ migration). We measured no changes in the surface expression of activation markers CD80, CD86 and CD25; the endocytic receptors CD206 (Mannose receptor) and CD209 (DC-SIGN) or the regulatory markers CD200R, PD-L1 and Immunoglobulin-like transcript 3 (ILT3; Fig 3A) at a recCSP dose of 250ng/ml. Increasing the dosage of recCSP had no effect (2.5–2500 ng/ml; S2A Fig) nor did recCSP modulate LPS-induced activation of MoDCs (S2B Fig). In line with this, co-culturing of LPS-matured, recCSP-stimulated MoDCs with naïve CD4^+^ T cells did not result in polarization of T cells to a Th1 (IFNγ), Th2 (IL-4) or regulatory (IL-10) response (Fig 3B). Similarly to the MoDCs, MoMϕs did not change their phenotype upon recCSP stimulation (Fig 4; S4 Fig). Overall, these data show that MoDCs and MoMϕs do not respond phenotypically to recCSP stimulation and uptake.

### Uptake of Pf sporozoites activates MoMϕs in vitro

Next, we tested APC responses to whole *Pf* SPZ stimulation. SPZ stimulation of MoDCs did not result in upregulation of surface activation markers, and did not alter their capacity to prime effector CD4^+^ T cells or regulatory T cells (S3 Fig), indicating that SPZ stimulation of MoDCs has no effect on their phenotype or function.

In contrast, SPZ stimulation of MoMϕs resulted in an increased surface expression of activation markers (CD80 and CD25) as well as regulatory markers (PD-L1 and ILT3), which was not seen with uninfected salivary gland extract (SGE) alone (Fig 4A; S4 Fig). This phenotype of mixed pro- and anti-inflammatory surface marker expression was mirrored in cytokine production, where we saw approximately 5-fold increases of pro-inflammatory IL-6 and IL-1β, together with a more pronounced 12-fold increase in hallmark regulatory cytokine IL-10 upon stimulation with SPZ. This enhanced production was not seen in MoMϕs stimulated with recCSP and was much lower with SGE alone (Fig 4B; S5 Fig). Opsonization of SPZ did not significantly influence their surface marker responses, although there was a trend towards increased activation and a concomitant decrease in regulatory markers (Fig 4A). Interestingly, opsonization of SPZ abrogated IL-1β production by MoMϕs (Fig 4B), although titers of IL-1β were significantly lower than those of IL-6 and IL-10. Overall, these data show MoMϕs are activated by *Pf* sporozoite stimulation, resulting in a mixed pro- and anti-inflammatory phenotype.

### MoMϕs display decreased motility after SPZ stimulation

**Fig 5:**
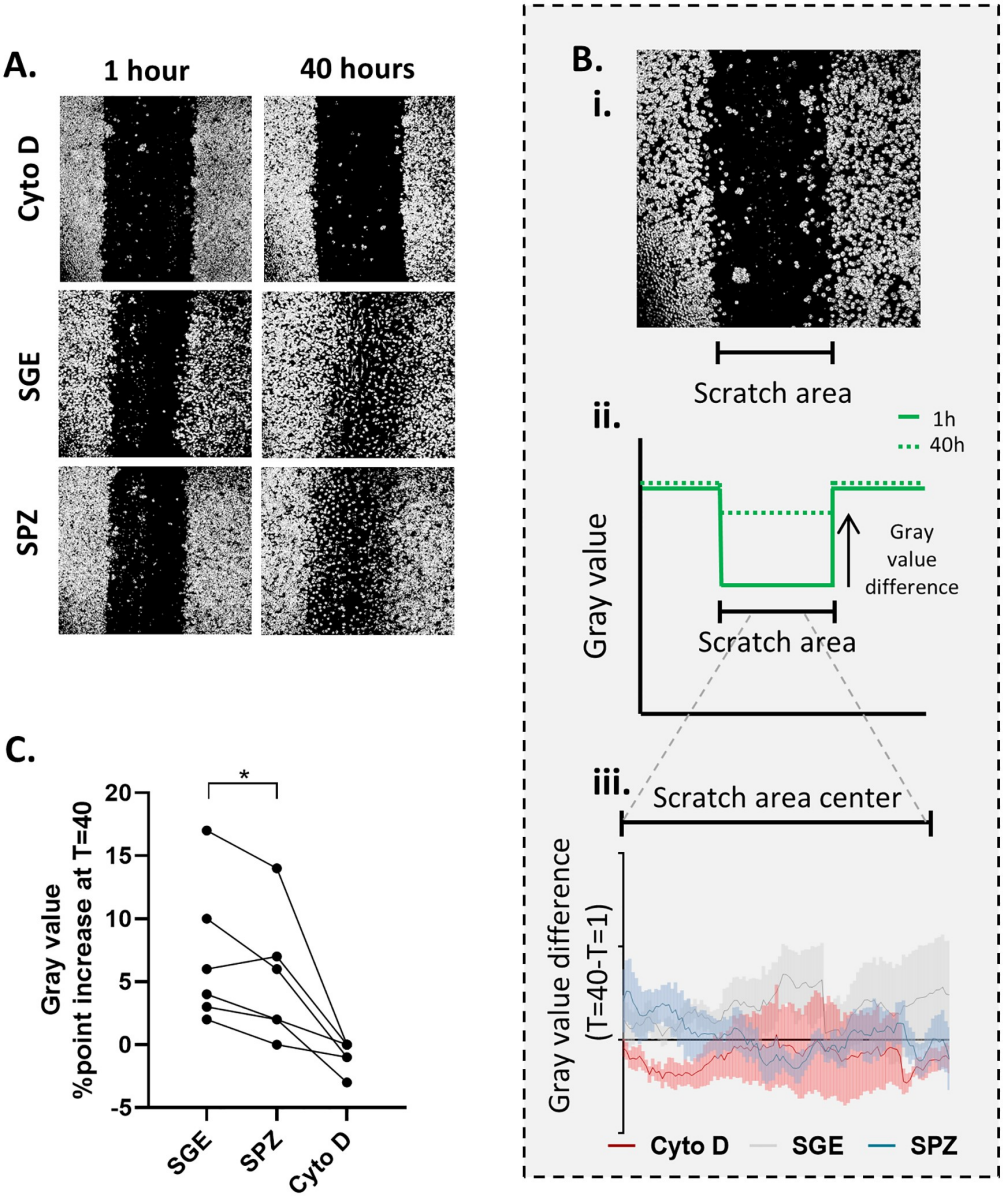
MoMϕ migration upon *Pf* SPZ stimulation. **A**. Representative examples of wound closing assay. The images show wells containing MoMϕs (grey) with a central “wound” (black), where MoMϕs have been scratched away. After 40 hours, blurring of the scratch indicates migrated MoMϕs. Cytochalasin D (Cyto D) blocks all cell migration as control. **B. i**. example of a scratch with indication of scratch area to be analyzed. **ii**. Schematic of a surface plot of entire microscopic image. An increase in the gray value is seen over the scratch area over time, as cells fill up the wound. **iii**. Difference in gray value between T = 40 and T = 1 over the scratch area center, where an increased difference reflects higher migration. Lines indicate the mean of three independent experiments including six donors. Line width indicates Standard Error of the Mean (SEM). As cells migrate from the outside inward, a decrease in migration is seen primarily in the center of the scratch area. *Pf* SPZ stimulated MoMϕs display reduced wound closure after 40 hours. Statistical analysis using two way ANOVA with Tukey test for multiple comparison, SPZ compared to SGE p = <0.0001. **C**. Mean percent point difference in gray value over the scratch area (T = 40-T = 1) over time per donor. Analysis using paired student’s T test *: p = <0.05.

The combined increased surface expression of activation markers as well as regulatory markers on macrophages has been described previously in tumor immunology, where they are referred to as regulatory macrophages, or Mregs. Tumor-associated macrophages have been shown to exhibit decreased motility upon exposure to cancer cell products[53]. To investigate whether SPZ have a similar effect on MoMϕs, we performed a wound closing assay in which MoMϕs were allowed to migrate for 40 hours after disturbance of their monolayer in a cell culture plate (“wounding” by scratch). As a control we used Cytochalasin D (Cyto D), which blocks migration by inhibition of actin polymerization[51]. MoMϕs stimulated with SPZ displayed reduced wound closure at 40 hours compared to MoMϕs stimulated with SGE alone (Fig 5B and C; p = <0.0001; S6 Fig). Therefore, SPZ stimulated MoMϕs seem to share multiple characteristics with tumor Mregs, including the molecules they express on the surface and their motility.

### SPZ-stimulated MoMϕs decrease IFNγ production during DC-mediated CD8^+^ T cell priming

**Fig 6:**
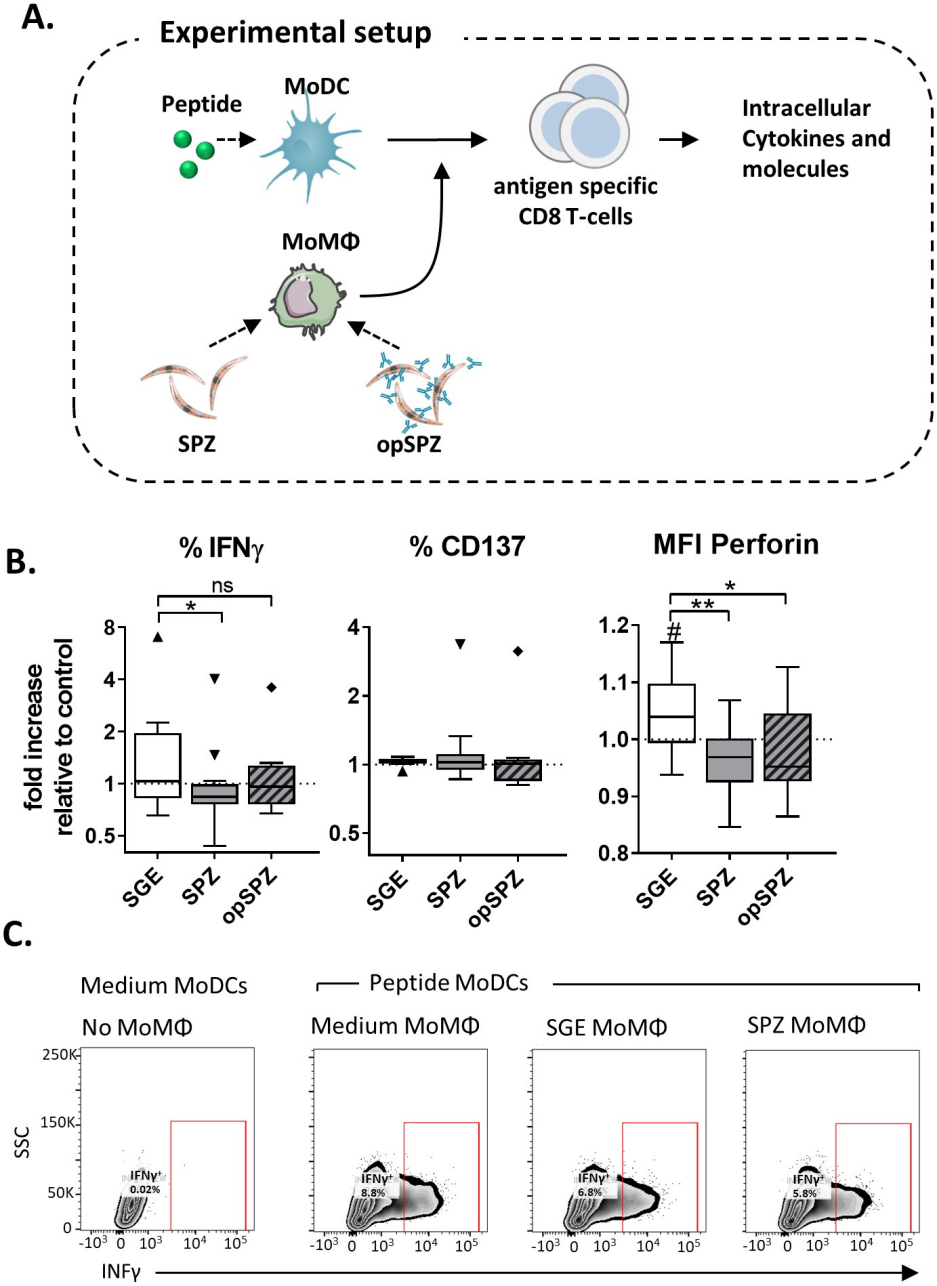
Immune suppression by *Pf* SPZ stimulated macrophages. **A**. Experimental setup. Peptide stimulated MoDCs are co-cultured with a peptide specific CD8^+^ T cell clone in the presence or absence of parasite or control stimulated MoMϕs (information in materials and methods). **B**. CD8 ^+^ cell phenotype after overnight co-culture with MoDCs and MoMϕs. Intracellular IFNγ, activation marker CD137 and Perforin expression. Analysis using Wilcoxon test, N = 4, 8 donors. #: P = <0.05 compared to medium control. *: P = <0.05, **: P = <0.005. **C**. Representative cytometry plots showing percentage of IFNγ^+^ CD8 ^+^ T cells.

Next, we explored whether the regulatory phenotype of SPZ-stimulated MoMϕs allows them to suppress CD8^+^ T cell responses. MoMϕs stimulated with SPZ, SGE lone or LPS were co-cultured with an antigen-specific CD8^+^ T cell clone in the presence of antigen-loaded MoDCs (Fig 6A). Intracellular cytokine analysis revealed that fewer CD8^+^ T cells produced IFNγ in the presence of MoMϕs stimulated with SPZ (mean reduction 21% compared to SGE; P = <0.05) and produced less perforin (Fig 6B and S7 Fig; mean reduction 9% compared to SGE; p = 0.01). Opsonized SPZ were not as effective in reducing IFNγ production by CD8^+^ T cells as compared to un-opsonized SPZ (Fig 6B and S7 Fig; mean reduction 6% IFNγ compared to SGE), but were equally capable of reducing perforin responses. We found no changes in granzyme A and B production by the T cell clone in the presence of MoMϕs stimulated with SPZ (S7 Fig). Together, these data confirm a modest functional regulatory potential of SPZ-stimulated MoMϕs.

**Fig 7:**
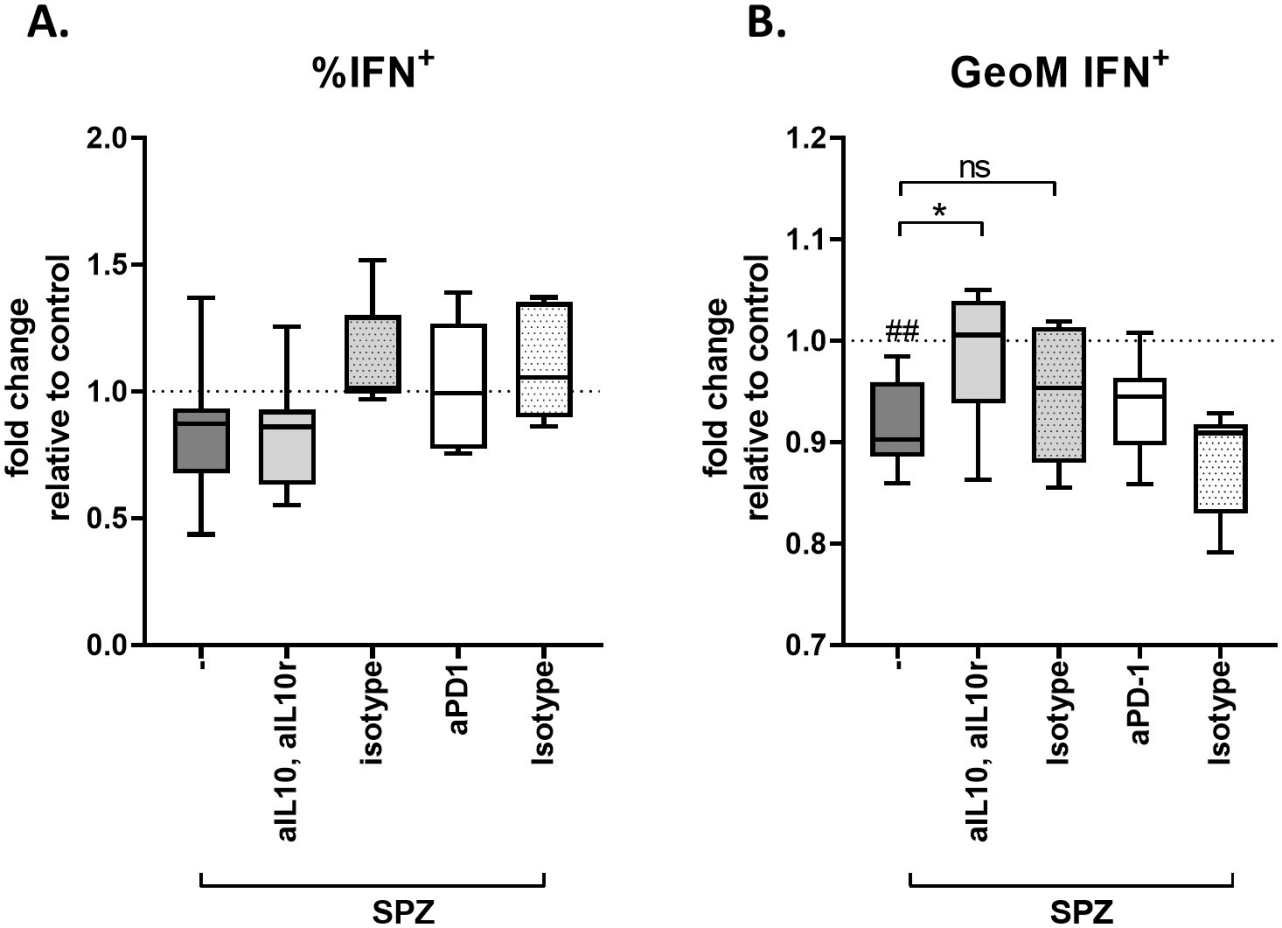
Blocking the IL10 pathway partially restores IFNγ production in IFNγ^+^ CD8^+^ T cells. **A**. Blocking IL-10 and its receptor, or PD-1 does not restore the percentage of IFNγ producing CD8 T cells. Data shows % of IFNγ^+^ antigen specific CD8^+^ T cells after co-culture with antigen pulsed MoDCs in the presence of macrophages stimulated with *Pf* SPZ alone (-) or *Pf* SPZ and anti IL-10(R) or PD-1 antibodies or their isotype controls. **B**. Blocking IL-10 and its receptor, but not PD-1 restores the levels of IFNγ produced by IFNγ^+^ CD8 T cells (data shown in Mean Fluorescent intensity compared to medium control). # indicates comparison to control (standardized to 1). ##: p = <0.01, *: p = <0.05.

Next we investigated whether IL-10 or PD-L1 play a role in the induction of the immunosuppressive capacity of SPZ stimulated MoMϕs by blocking the IL-10 or PD/PD-L1 pathway using antibodies against IL-10 and IL-10 receptor (IL10R) or PD-1 respectively. Blocking of the IL-10 or PD-L1 pathway did not restore the percentage of CD8 T cells producing IFNγ (Fig 7A). However, blocking IL-10 and IL-10R, but not PD-1, restored the mean fluorescence intensity (MFI) of IFNγ in IFNγ -producing CD8+ T cells, indicating that the suppression of CD8^+^ T cell responses by MoMϕs is at least partially IL-10 dependent (Fig 7B).

### Human dermal APCs responses to SPZ mimic MoMϕ responses

**Fig 8:**
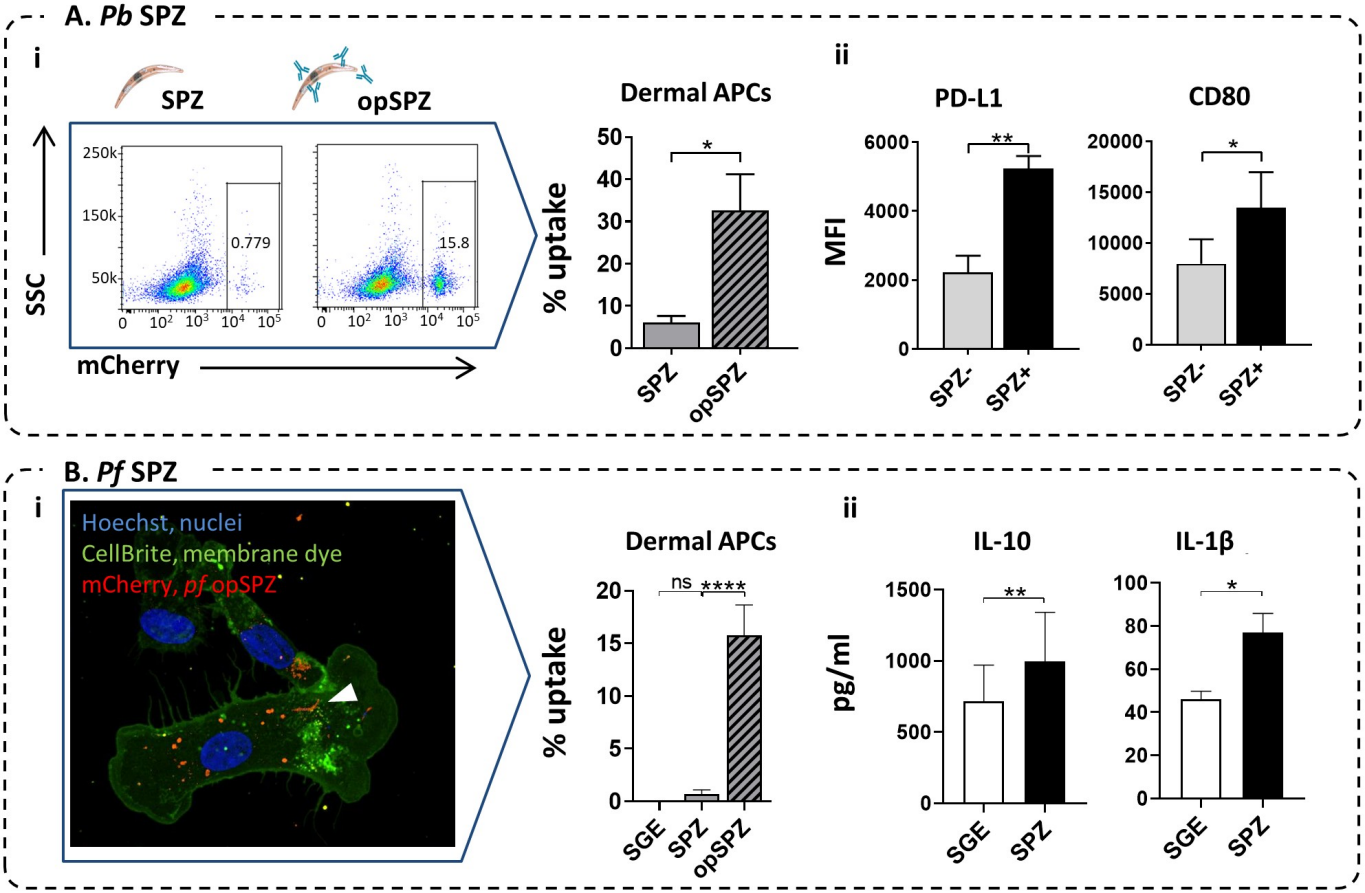
Human skin APC responses to SPZ. **A. i**. *Pb* SPZ uptake in dermal APCs (HLA-DR^+^, CD11c^+^) from lysed skin explants. Representative plots and quantification of flow cytometric results. N = 4, 6 donors. **Ii**. PD-L1 and CD80 expression in lysed dermal APCs in the SPZ^+^ (after uptake of *Pb* opSPZ) or SPZ^-^ subset. N = 3, 3 donors. **B. i**. *Pf* SPZ uptake in dermal APCs (emigrated from human skin explants) Representative image and quantification of confocal microscopy results. N = 3, 3 donors. **ii**. IL-10 and IL-1β production by *Pf* SPZ or SGE stimulated lysed skin cells. *: p = <0.05, **: p = <0.01, ****: p = <0.0001. N = 3, 4 donors.

To confirm that human skin APCs respond in a similar fashion as their monocyte-derived counterparts, we stimulated primary dermal APCs obtained from human skin explants with *Pb* and *Pf* SPZ or SGE *in vitro*. Approximately 6% (range 1.4–10.7) of primary HLA-DR^+^, CD11c^+^ total dermal APCs (this population contains both dermal Mϕ and DC populations as surface marker plasticity impairs distinguishing dermal Mϕ from dermal DCs) took up *Pb* SPZ. When SPZ were opsonized, the uptake increased five-fold to 33% (range 11.4–61.7), mimicking MoMϕs responses to SPZ opsonization (Fig 8A i). In line with this, confocal quantification of *Pf* SPZ uptake in dermal APCs showed 0.7% uptake of *Pf* SPZ on average. This increased to 15% after opsonization of SPZ (Fig 8Bi).

In addition, corroborating MoMϕ responses, phenotypic responses of dermal APCs after uptake of *Pb* SPZ was characterized by gating on SPZ^+^ cells and revealed increased surface PD-L1 as well as CD80 expression upon phagocytosis of *Pb* parasites (Fig 8Aii). Total dermal APC surface PD-L1 and CD80 did not show significant differences between SGE and *Pf* SPZ stimulation (S8 Fig). Flow cytometric detection of SPZ uptake was not technically feasible with *Pf*, therefore we analyzed the supernatant of these cells for IL-10 and IL-1β production. Paralleling MoMϕ responses, IL-10 and IL-1β production increased upon *Pf* SPZ stimulation of dermal APCs. Collectively, these data indicate that dermal primary APC responses resemble MoMϕ responses to SPZ, and indicate a regulatory role upon encounter with SPZ.

## Discussion

Here we show that malaria SPZ induce Mϕs which express PD-L1, produce IL-10 and show reduced motility. In addition, these Mϕs suppress IFNγ and perforin production by memory CD8^+^ T cells. This early-onset suppression of host defenses by SPZ at the skin stage may help explain why immunity to sporozoites is difficult to induce and can be highly relevant for improving next generation CSP-based or attenuated SPZ vaccines.

Interestingly, these Mϕs retained their ability to produce IL-6 and express CD80. The production of regulatory IL-10 and the expression of inhibitory PD-L1 and/or PD-L2[54], despite their retained ability to produce proinflammatory cytokines and express high levels of co-stimulatory molecules[55] is characteristic of regulatory Mϕs (Mregs). Mregs have primarily been described in the physiological context of tissue repair[54] and pathological context of cancer[56], where they promote wound healing and tumor outgrowth respectively. However, in chronic parasitic diseases such as leishmania[57] and trypanosoma[58] as well as helminths[37, 59, 60] these monocytes/macrophages have also been suggested to play a role in immune evasion. With regard to helminths, we have previously shown that *Schistosoma mansoni*, the causative agent of Schistosomiasis and also a skin-penetrating parasite, induces a similar early dermal induction of immune regulation, characterized by PD-L1 expressing and IL-10 producing dermal APCs[35]. The finding that Mregs could play a role in immune evasion by malaria in addition to other parasitic diseases provides compelling support for the evaluation of checkpoint inhibitors in these chronic infections[61].

Our data did not show any phenotypic changes of both MoDCs and MoMϕs in response to recCSP. CSP is the most abundant SPZ antigen in the pre-erythrocytic stage of malaria and has been shown to be sufficient for inducing proliferation of CSP-specific CD8^+^ T cells *in vitro*[62].

We used a commercially available recCSP which contains a large part of the repeat region (B-cell epitopes), the full C terminal containing the majority of known T-cell epitopes and the glycosylphosphatidylinositol (GPI) anchor sequence [63, 64], similar to the CSP protein sequence used in the CSP-based malaria vaccine RTS,S. These recombinant CSPs lack the native parasite GPI anchor which is a known Toll-like receptor (TLR)-2/4 activating pathogen-associated molecular pattern (PAMP) in protozoa[65] and plays a role in blood stage malaria[66]. We conclude that despite containing the B- and T-cell epitope regions and the GPI anchor sequence, recCSP alone is not sufficient to trigger an innate immune response. This may explain why strong adjuvants such as GlaxoSmithsKline’s Adjuvant System are needed to induce sufficient antibody levels in the CSP-based malaria vaccine RTS,S[67]. In the setting of natural infection, danger associated molecular patterns (DAMPS) introduced via cell damage by mosquito probing and/or parasite migration through the skin could provide necessary stimulation of APCs to induce activation. Alternatively, the low immunogenic potential of the antigen alone may be critical to the survival of the parasite as it would initiate T cell anergy in the absence of co-stimulation, or the induction of regulatory T cells.

In addition to antibody responses, CD8^+^ T cells play a central role in the protection against pre-erythrocytic stages of malaria[8, 68, 69]. Clinical trials have shown that sterile protection largely correlates with total numbers of IFNγ producing CD8^+^ T cells[7]. Next to IFNγ, other cytotoxic molecules produced by CD8^+^ T cells such as perforin and granzymes have also been associated with protection[70]. We show that SPZ-stimulated MoMϕs can suppress IFNγ and perforin, but not granzyme production by MoDC-activated CD8^+^ T cells, underlining the potentially detrimental effect of regulatory Mϕs on immune responses in the context of attenuated SPZ vaccines. Because blocking IL-10 and its receptor only partially restored IFNγ production, additional interactions between macrophages, dendritic cells and CD8+ T cells are likely to add to the observed immune-suppressive effect.

In order to translate our findings to the human skin setting, we used primary human dermal APCs alongside monocyte-derived APCs and showed that the primary cells exhibit a similar phenotype compared to MoMϕs. Where the priming of CD8^+^ T cells subsequently takes place after attenuated SPZ vaccination is an ongoing matter of debate[71, 72]. Given the nature of macrophages and a further reduced motility, it seems unlikely that SPZ-induced regulatory macrophages migrate to regional lymph nodes. Potentially, regulation could be established directly in peripheral tissues, by affecting dermal-resident T cells or T cells recruited to the site of infection. Alternatively, regulatory signals may be conveyed to dermal DCs[73], thereby influencing the priming of CD8^+^ T cells. Lastly, SPZ have been shown to migrate out of the skin site to local lymph nodes, where they could induce a similar tolerogenic response by LN-resident macrophages[74]; similar mechanisms could potentially be exploited in liver tissue via liver-resident macrophages, Kupffer cells[75]. A translation to *in vivo* models, such as controlled human infections[76, 77], or the use of primate models for malaria (with skin more comparable to that of humans) will allow for a more detailed dissection of the site of immune cell interaction and cell types involved.

We revealed a dual effect of SPZ antibodies on APC responses: As non-opsonized SPZ induced a regulatory phenotype in APCs, boosting the efficiency of SPZ phagocytosis could result in an increased number of Mregs. However, phenotypic analysis showed a trend towards decreased regulatory markers when SPZ were pre-incubated with a monoclonal anti-CSP antibody and opsonization also restored CD8^+^ T cell IFNγ production. These findings are in line with previous reports that Fc gamma receptor (FcγR) stimulation promotes proinflammatory responses[78, 79]. Whether similar effects would occur in the presence of polyclonal anti-SPZ antibodies remains to be investigated. How this would ultimately translate to the *in vivo* situation is unclear, as factors such as reduced SPZ motility play an important role in the local tissue density of Mϕs that have interacted with SPZ[80], as well as the local immune (regulatory) environment. Understanding the interplay between antibodies and ensuing immune responses has important implications for vaccine development. For example, pre-existing anti-CSP antibodies[81, 82] in endemic populations may neutralize vaccine SPZ through opsonization and increased phagocytosis. However, anti-CSP antibodies can be used as a surrogate of protection in vaccine studies involving RTS/S[83, 84]. Taken together, this underlines the need to fully appreciate the net effect of natural and vaccine boosting in pre-erythrocytic vaccine strategies.

To date, dermal immune responses to malaria and vaccine candidates are wholly uncharacterized in humans, yet the development of an effective live pre-erythrocytic malaria vaccine arguably is crucially dependent on our understanding of these initial parasite-host interactions. Whether the relatively minor differences in CD8 T cell function have an impact on overall immunity to malaria during natural infection, where SPZ concentration in the skin is much lower than in our *in vitro* assays, remains to be determined. With this study, we aimed to elucidate the critical initial steps of human skin stage malaria. Such insights into the immune regulatory properties of SPZ are a pivotal first step in broadening our understanding of pre-erythrocytic natural immunity and the pitfalls of intradermal vaccination-induced immunity.

## Supporting information

S1 Movie MoMΦs phagocytize opsonized *Pf* SPZ in vitro. (MP4)

S2 Movie MoDCs do not interact with whole *Pf* SPZ in vitro. (MP4)

S1 Fig *Pf* SPZ uptake cannot be analyzed by flow cytometry due to high autofluorescence of (monocyte-derived) APCs. A. *Pf* SPZ mCherry fluorescence signal by flow cytometry of mosquito salivary glands alone (top panels, SPZ on the left, SGE control on the right) and after stimulation of MoMϕs (bottom panels). Whereas SPZ mCherry signal can be easily distinguished when measuring SPZ in salivary gland samples, MoMϕs show high autofluorescence in mCherry. **B**. Histogram of mCherry fluorescence in MoMϕs stimulated with opsonized *Pf* SPZ (blue) or SGE control (red).
(TIF)

S2 Fig MoDC responses to recCSP. **A**. Surface marker responses to increasing doses of recCSP on immature MoDCs. **B**. Surface marker responses to increasing doses of recCSP on LPS matured MoDCs. Data shown as fold changes in Median Fluorescence Intensity (MFI) compared to medium stimulated controls (dotted line). N = 8, at least 10 donors.
(TIF)

S3 Fig MoDC responses to *Pf* SPZ. **A**. MoDC surface marker expression after stimulation with SGE, *Pf* SPZ, opsonized *Pf* SPZ (opSPZ) or LPS control. Data shown in MFI (median fluorescence intensity) relative to unstimulated MoDC control. Data of 1 experiment, 3 donors. **B-C**. CD4^+^ T cell polarization after *Pf* SPZ stimulation. *Pf* SPZ stimulation of (LPS-matured) MoDCs does not polarize naïve T cells towards a Th1 (IFNγ) or Th2 response (IL-4; both measured by intracellular staining. (Data shown relative to LPS-matured MoDC control, N = 3, 10 donors, soluble Schistosome Egg Antigen (SEA) used as a Th2 inducing control. **: P = <0.005.) **D**. CD4^+^ T cell **r**egulatory response (IL-10; measured by ELISA after CD3/28 restimulation) after coculture with *Pf* SPZ stimulated (LPS matured) MoDCs. Data shown relative to LPS-matured MoDC control. N = 3, 6 donors. **E**. MoDCs do not induce regulatory T cells in response to *Pf* SPZ. Data shown as the division index (average number of memory T cell divisions) of memory T cells after stimulation with LPS MoDCs in the presence of SPZ induced T cells, as calculated by FlowJo. Data from 1 experiment, 4 donors.
(TIF)

S4 Fig MoMΦ surface marker expression in response to recCSP or whole *Pf* SPZ. Surface marker expression of MoMΦs in response to stimulation with recCSP (250ng/ml), *Pf* SPZ and *Pf* opSPZ. **A**. SPZ stimulation induces increased activation marker expression (CD80, and CD25). No response to recCSP. **B**. SPZ stimulation reduces CD197 expression. No significant change in M2 markers CD206 and CD209. **C**. SPZ stimulation increases regulatory markers PD-L1, PD-L2 and ILT3. Data shown as fold changes in Median Fluorescence Intensity (MFI) compared to medium stimulated controls (dotted line). # indicates analysis compared to medium control. #: P = <0.05, ##: P = <0.005, ###: P = <0.0005 and ####: P = <0.0001, * = P<0.05, ** = P<0.001, *** = P<0.005, **** = P<0.0001 using one way ANOVA. N = 9, at least 20 donors.
(TIF)

S5 Fig MoMΦ responses to recCSP and whole SPZ. Cytokine Il-10, IL-6 and IL1β responses to recCSP and *Pf* SPZ stimulation. Data shown in pg/ml; IL-10: N = 8, 25 donors; IL-6: N = 5, 17 donors; IL1β: N = 3, 7 donors.
(TIF)

S6 Fig MoMΦ cell death *in vitro* over time. **A**. Mean gray value measured over the sides on both side of the scratch show no differences in cell density over time between or within groups, indicating the reduced gray value over the scratch is not due to overall cell loss. **B**. % of Live MoMϕs at 1 and 40h of stimulation with SGE, *Pf* SPZ and Cyto D, measured by Flow Cytometry. Statistical testing using two way ANOVA. *p = <0.05 Although Cyto D reduces cell viability compared to SGE or *Pf* SPZ stimulation, we found no differences in cell viability of SGE and SPZ stimulated MoMϕs over time. N = 2, 5 donors.
(TIF)

S7 Fig CD8 responses are altered by SPZ stimulated MoMϕ. IFNγ, CD137 and perforin CD8^+^ T cell responses to control peptide stimulated MoDCs in the presence of SGE, *Pf* SPZ or *Pf* opSPZ stimulated MoMϕs. +CTRL represents response to peptide stimulated MoDCs in the presence of peptide stimulated MoMϕs. -CTRL represents baseline response to unstimulated MoDCs. Data shown as fold changes compared to peptide stimulated MoDCs only, in the absence of MoMϕ. IFNγ and CD137 responses shown as a percentage of total CD8 T cells. Perforin and Granzyme responses shown as Median Fluorescence Intensity (MFI). # indicates analysis compared to medium control. #: P = <0.05, ##: P = <0.005, using Wilcoxon test. N = 4, 8 donors.
(TIF)

S8 Fig Surface CD80 and PD-L1 expression of dermal APCs from lysed human skin explants after stimulation with *Pf* SPZ or SGE control. Mean fluorescence intensity of CD80 and PD-L1 in dermal APCs from lysed human skin cells (HLA-DR^+^, CD11c^-^) stimulated with SGE or *Pf* SPZ. N = 3, 4 donors.
(TIF)

S1 Table Excel spreadsheet of all figure data. (XLSX)
