# Supplementary figures and images for: Plasmodium sporozoites induce regulatory macrophages
Source: PLoS Pathog. 2020 Sep 8;16(9):e1008799. doi: 10.1371/journal.ppat.1008799 (PMC7500643; doi:10.1371/journal.ppat.1008799)

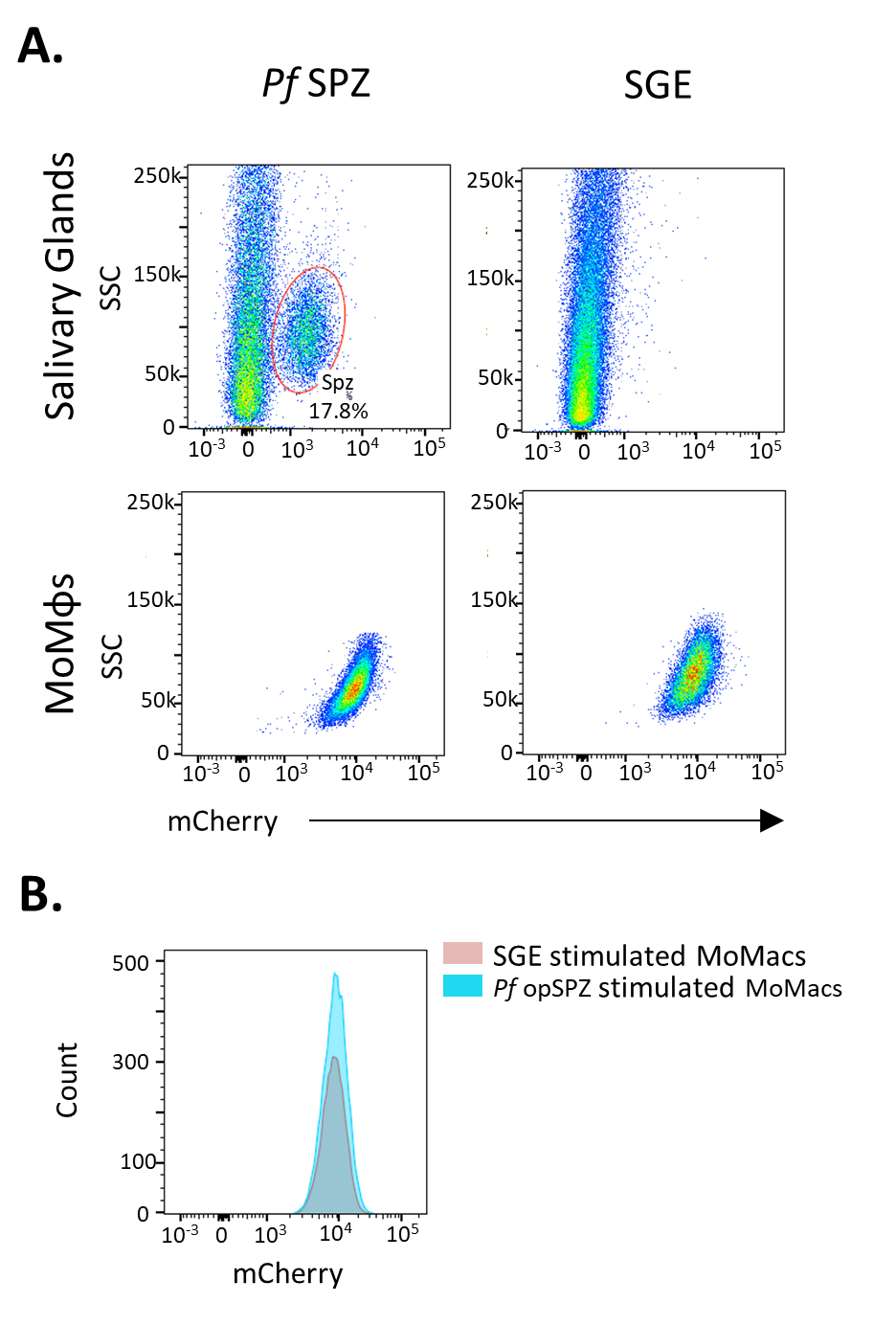

Supplement: S1 Fig — A. Pf SPZ mCherry fluorescence signal by flow cytometry of mosquito salivary glands alone (top panels, SPZ on the left, SGE control on the right) and after stimulation of MoMϕs (bottom panels). Whereas SPZ mCherry signal can be easily distinguished when measuring SPZ in salivary gland samples, MoMϕs show high autofluorescence in mCherry. B. Histogram of mCherry fluorescence in MoMϕs stimulated with opsonized Pf SPZ (blue) or SGE control (red). (TIF) [file ppat.1008799.s003.tif]

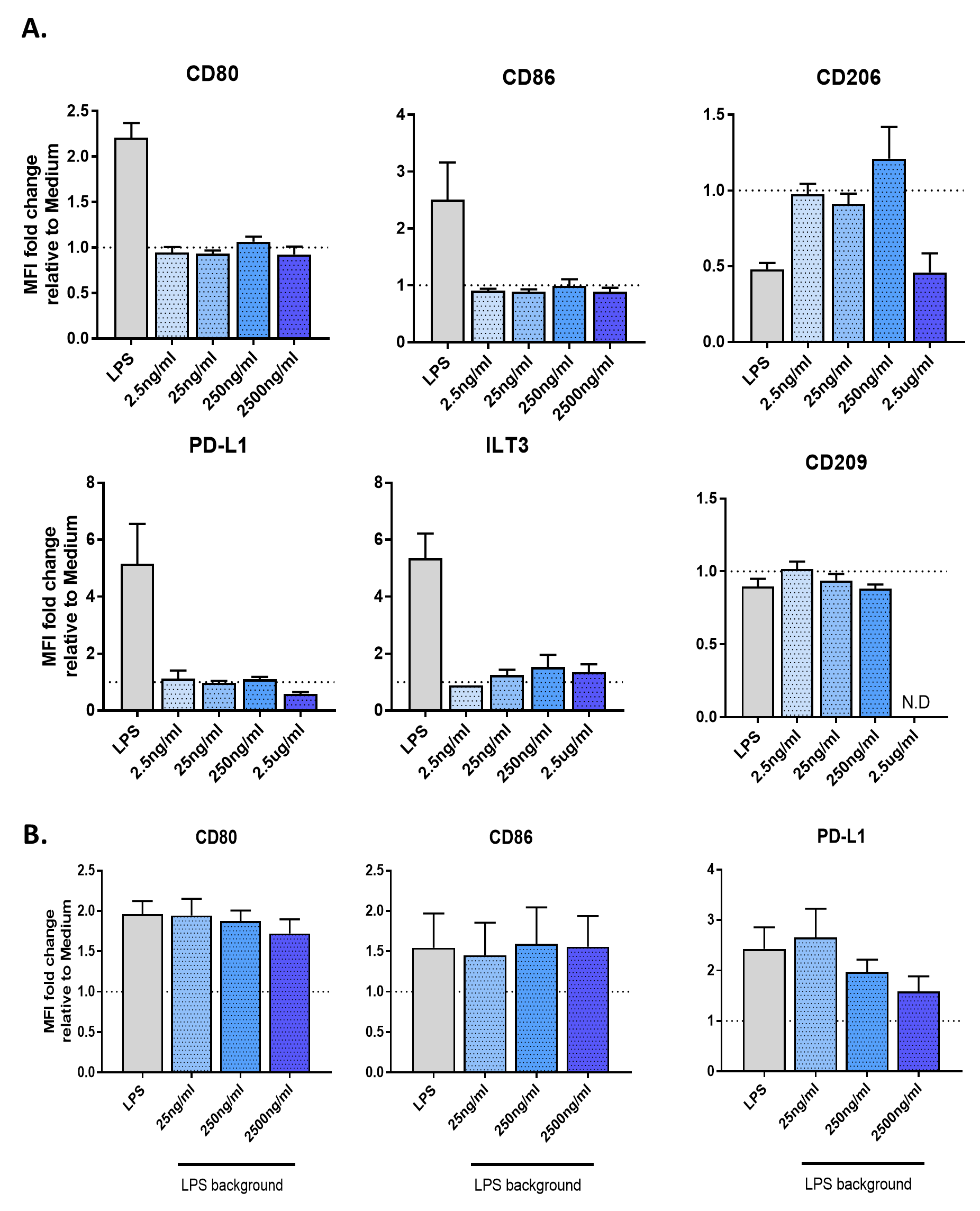

Supplement: S2 Fig — A. Surface marker responses to increasing doses of recCSP on immature MoDCs. B. Surface marker responses to increasing doses of recCSP on LPS matured MoDCs. Data shown as fold changes in Median Fluorescence Intensity (MFI) compared to medium stimulated controls (dotted line). N = 8, at least 10 donors. (TIF) [file ppat.1008799.s004.tif]

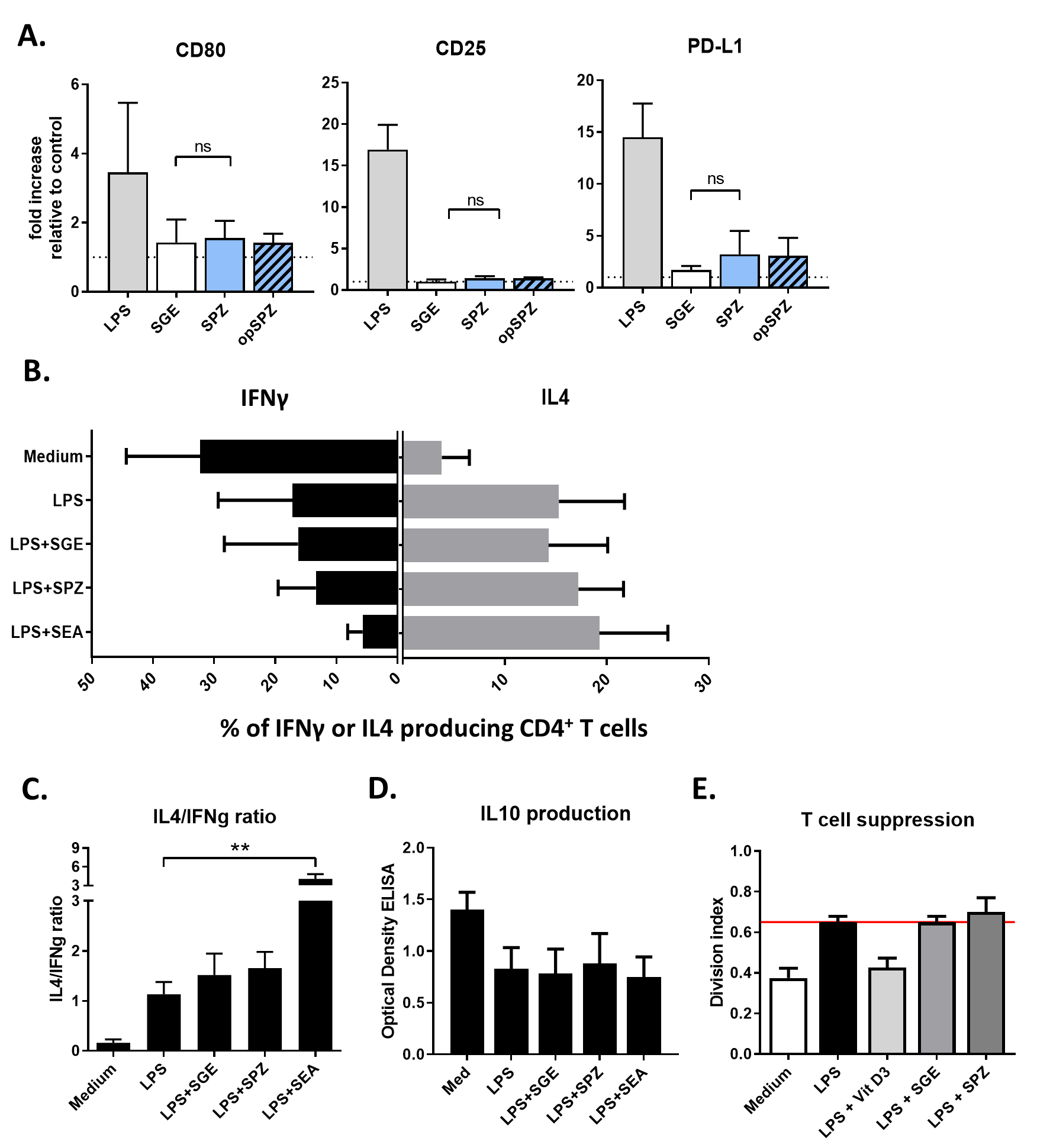

Supplement: S3 Fig — A. MoDC surface marker expression after stimulation with SGE, Pf SPZ, opsonized Pf SPZ (opSPZ) or LPS control. Data shown in MFI (median fluorescence intensity) relative to unstimulated MoDC control. Data of 1 experiment, 3 donors. B-C. CD4+ T cell polarization after Pf SPZ stimulation. Pf SPZ stimulation of (LPS-matured) MoDCs does not polarize naïve T cells towards a Th1 (IFNγ) or Th2 response (IL-4; both measured by intracellular staining. (Data shown relative to LPS-matured MoDC control, N = 3, 10 donors, soluble Schistosome Egg Antigen (SEA) used as a Th2 inducing control. **: P = <0.005.) D. CD4+ T cell regulatory response (IL-10; measured by ELISA after CD3/28 restimulation) after coculture with Pf SPZ stimulated (LPS matured) MoDCs. Data shown relative to LPS-matured MoDC control. N = 3, 6 donors. E. MoDCs do not induce regulatory T cells in response to Pf SPZ. Data shown as the division index (average number of memory T cell divisions) of memory T cells after stimulation with LPS MoDCs in the presence of SPZ induced T cells, as calculated by FlowJo. Data from 1 experiment, 4 donors. (TIF) [file ppat.1008799.s005.tif]

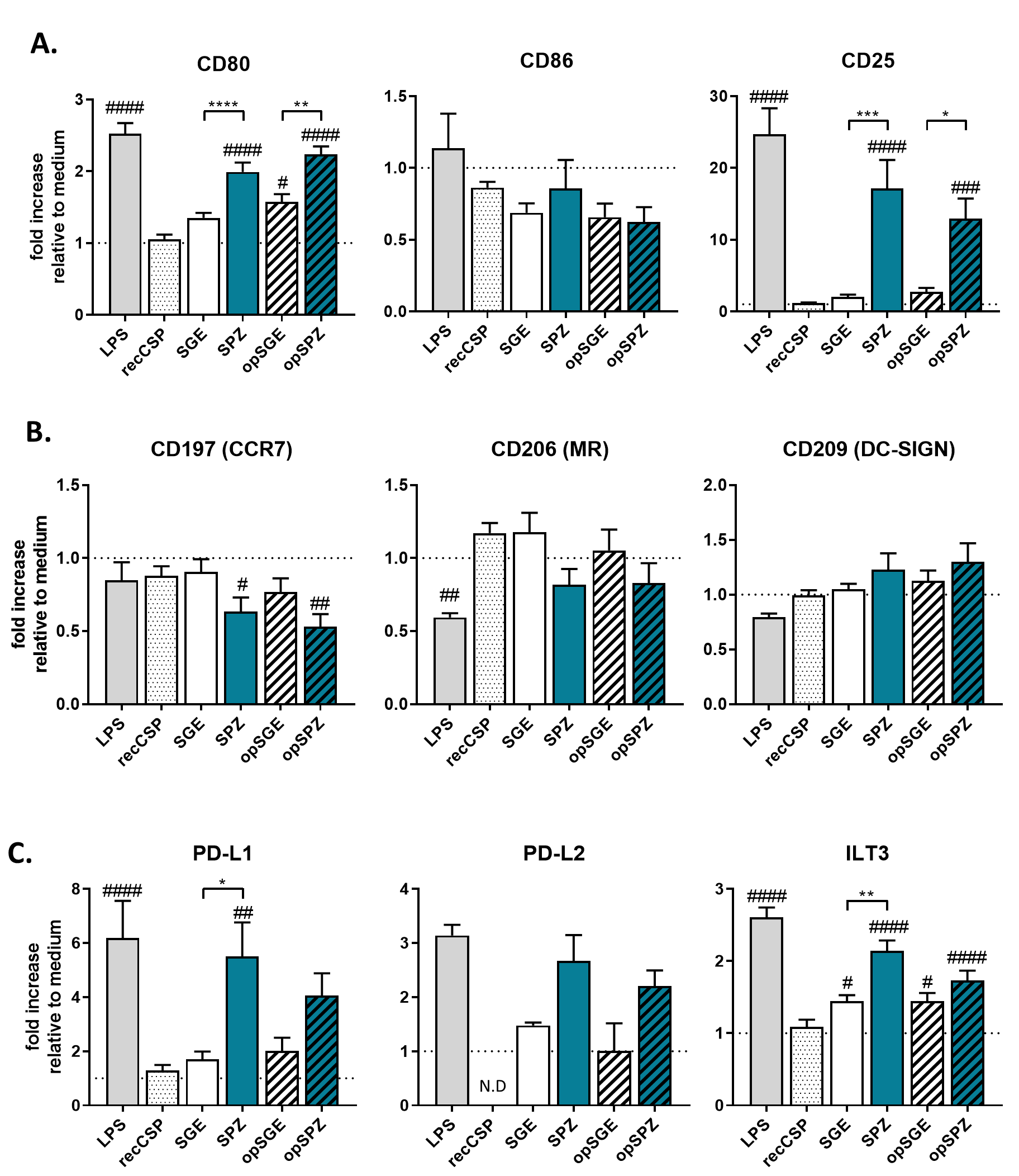

Supplement: S4 Fig — Surface marker expression of MoMΦs in response to stimulation with recCSP (250ng/ml), Pf SPZ and Pf opSPZ. A. SPZ stimulation induces increased activation marker expression (CD80, and CD25). No response to recCSP. B. SPZ stimulation reduces CD197 expression. No significant change in M2 markers CD206 and CD209. C. SPZ stimulation increases regulatory markers PD-L1, PD-L2 and ILT3. Data shown as fold changes in Median Fluorescence Intensity (MFI) compared to medium stimulated controls (dotted line). # indicates analysis compared to medium control. #: P = <0.05, ##: P = <0.005, ###: P = <0.0005 and ####: P = <0.0001, * = P<0.05, ** = P<0.001, *** = P<0.005, **** = P<0.0001 using one way ANOVA. N = 9, at least 20 donors. (TIF) [file ppat.1008799.s006.tif]

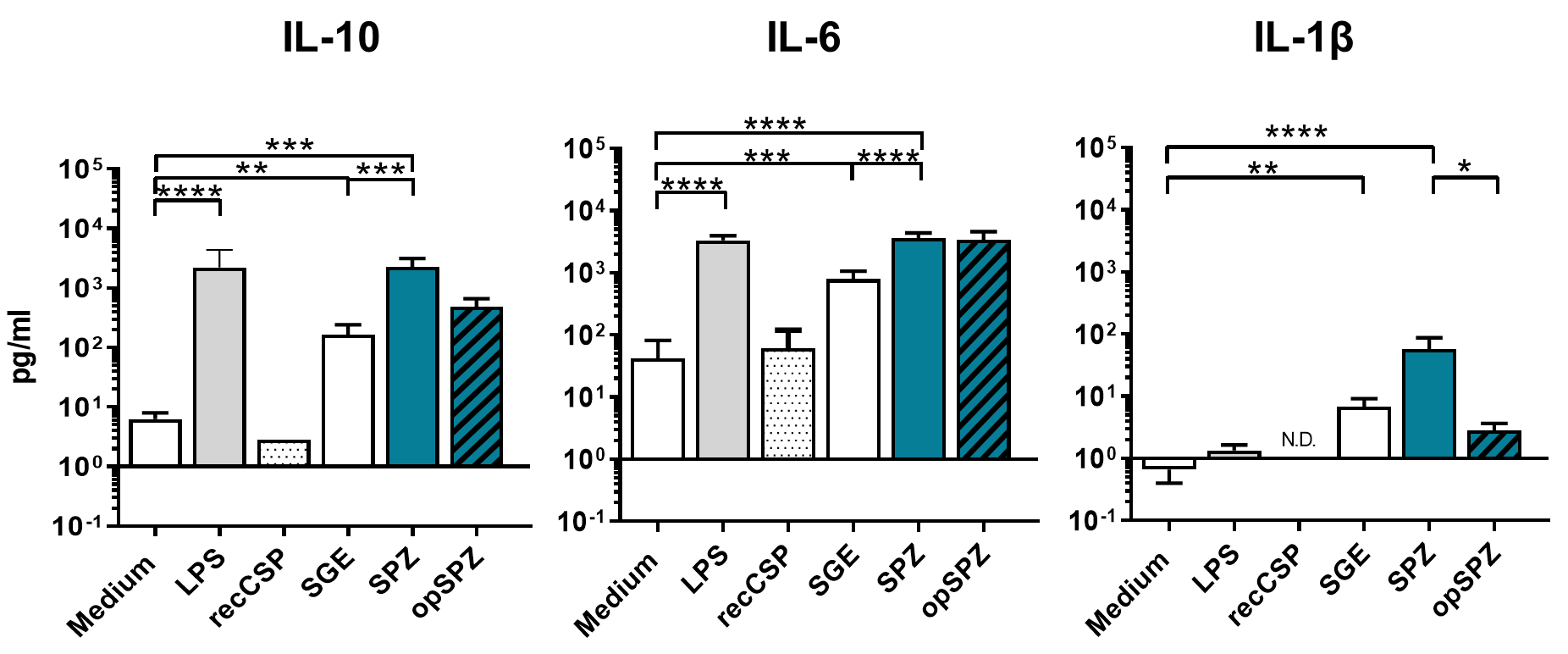

Supplement: S5 Fig — Cytokine Il-10, IL-6 and IL1β responses to recCSP and Pf SPZ stimulation. Data shown in pg/ml; IL-10: N = 8, 25 donors; IL-6: N = 5, 17 donors; IL1β: N = 3, 7 donors. (TIF) [file ppat.1008799.s007.tif]

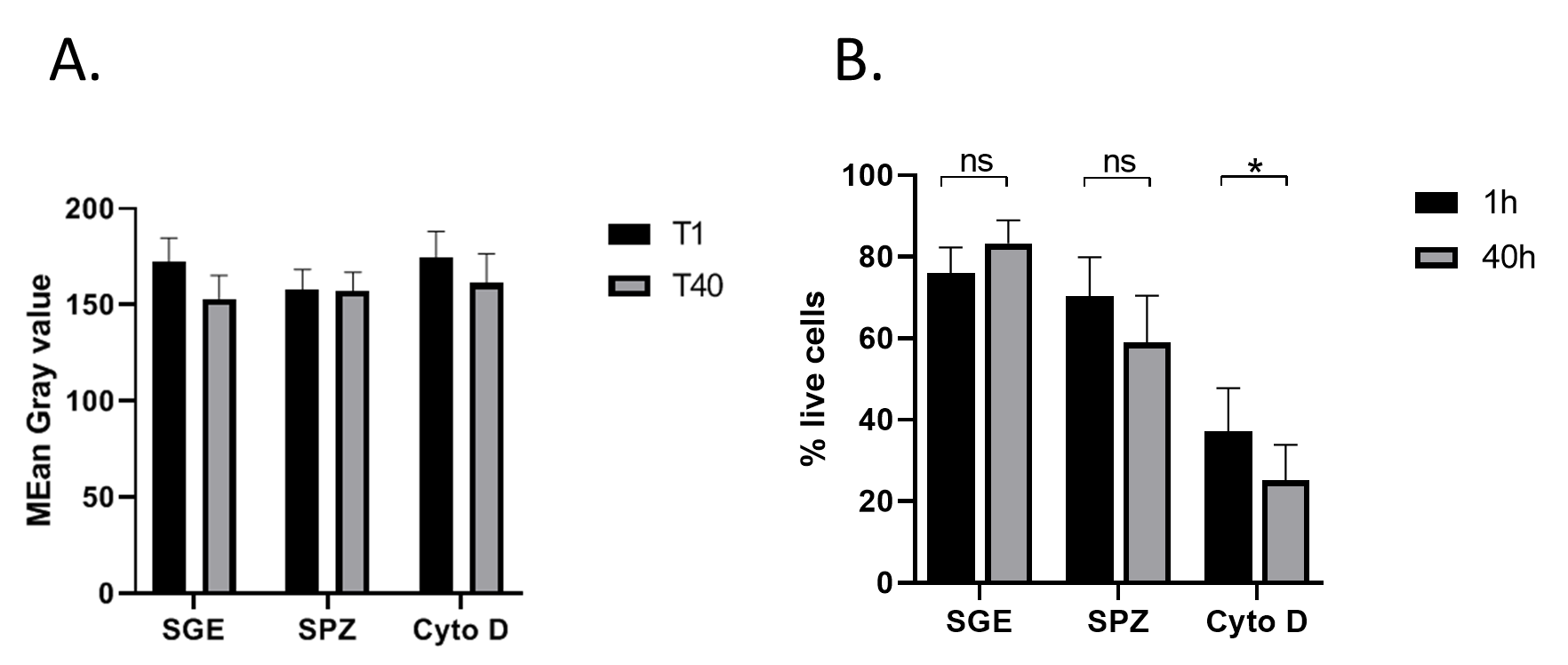

Supplement: S6 Fig — A. Mean gray value measured over the sides on both side of the scratch show no differences in cell density over time between or within groups, indicating the reduced gray value over the scratch is not due to overall cell loss. B. % of Live MoMϕs at 1 and 40h of stimulation with SGE, Pf SPZ and Cyto D, measured by Flow Cytometry. Statistical testing using two way ANOVA. *p = <0.05 Although Cyto D reduces cell viability compared to SGE or Pf SPZ stimulation, we found no differences in cell viability of SGE and SPZ stimulated MoMϕs over time. N = 2, 5 donors. (TIF) [file ppat.1008799.s008.tif]

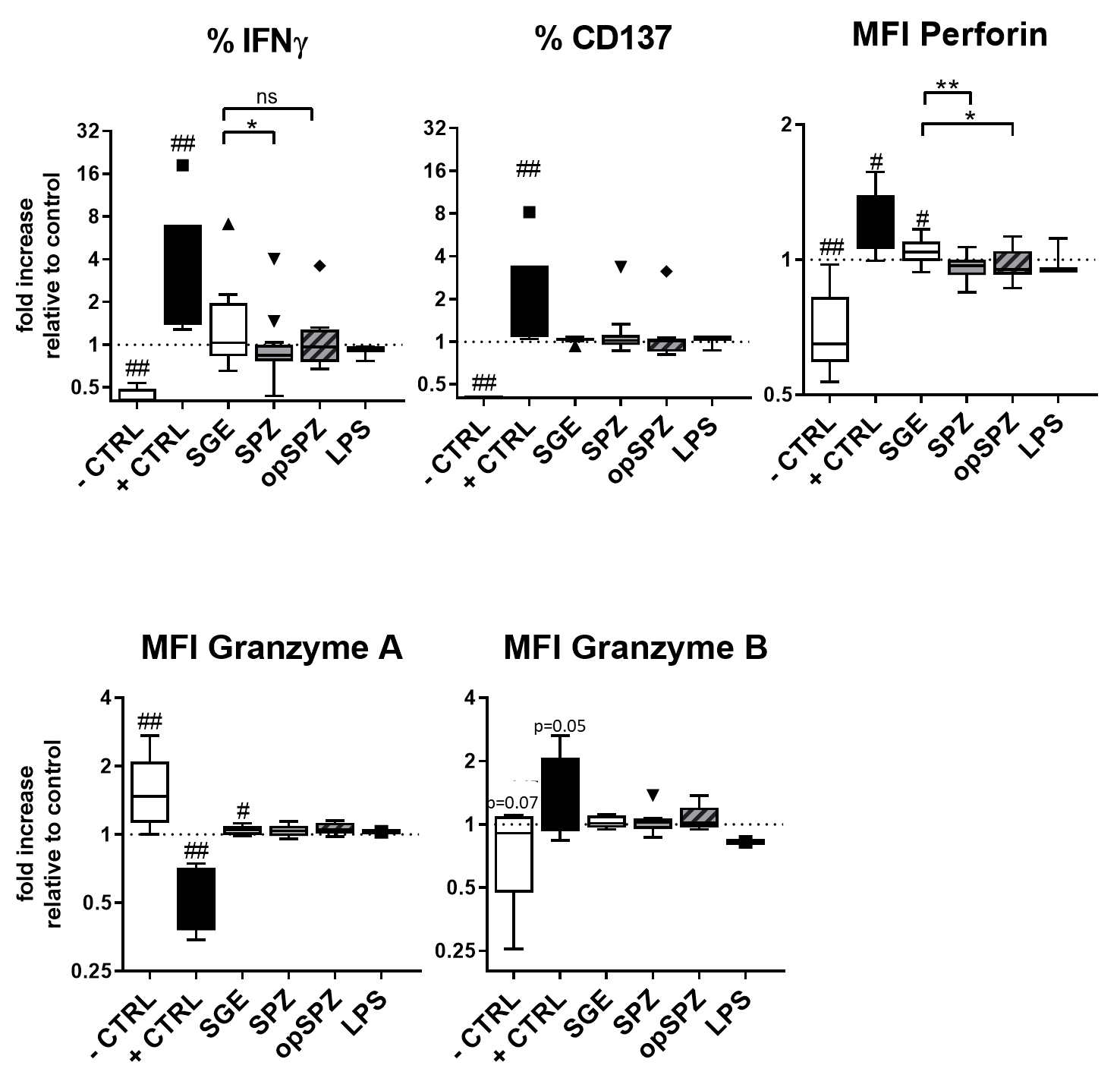

Supplement: S7 Fig — IFNγ, CD137 and perforin CD8+ T cell responses to control peptide stimulated MoDCs in the presence of SGE, Pf SPZ or Pf opSPZ stimulated MoMϕs. +CTRL represents response to peptide stimulated MoDCs in the presence of peptide stimulated MoMϕs. -CTRL represents baseline response to unstimulated MoDCs. Data shown as fold changes compared to peptide stimulated MoDCs only, in the absence of MoMϕ. IFNγ and CD137 responses shown as a percentage of total CD8 T cells. Perforin and Granzyme responses shown as Median Fluorescence Intensity (MFI). # indicates analysis compared to medium control. #: P = <0.05, ##: P = <0.005, using Wilcoxon test. N = 4, 8 donors. (TIF) [file ppat.1008799.s009.tif]

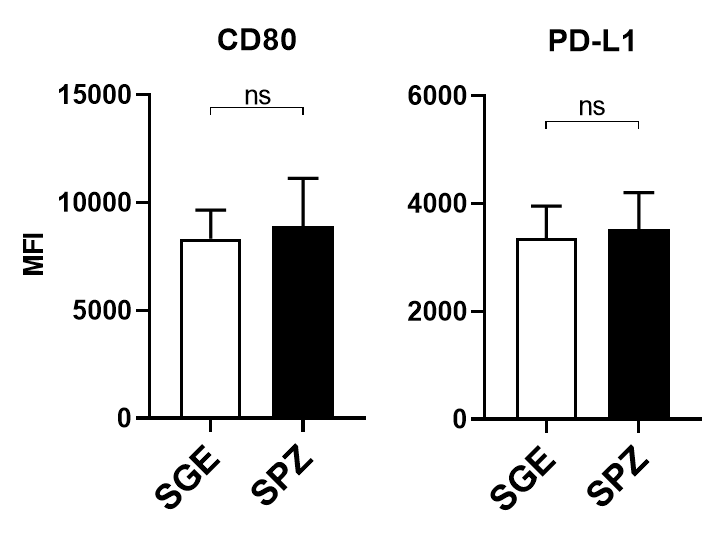

Supplement: S8 Fig — Mean fluorescence intensity of CD80 and PD-L1 in dermal APCs from lysed human skin cells (HLA-DR+, CD11c-) stimulated with SGE or Pf SPZ. N = 3, 4 donors. (TIF) [file ppat.1008799.s010.tif]
